# The non-canonical mechanism of ER stress-mediated progression of prostate cancer

**DOI:** 10.1186/s13046-021-02066-7

**Published:** 2021-09-14

**Authors:** Artem N. Pachikov, Ryan R. Gough, Caroline E. Christy, Mary E. Morris, Carol A. Casey, Chad A. LaGrange, Ganapati Bhat, Anatoly V. Kubyshkin, Iryna I. Fomochkina, Evgeniya Y. Zyablitskaya, Tatiana P. Makalish, Elena P. Golubinskaya, Kateryna A. Davydenko, Sergey N. Eremenko, Jean-Jack M. Riethoven, Amith S. Maroli, Thomas S. Payne, Robert Powers, Alexander Y. Lushnikov, Amanda J. Macke, Armen Petrosyan

**Affiliations:** 1 Department of Biochemistry and Molecular Biology, University of Nebraska Medical Center, Omaha, NE 68198 USA; 2 The Fred and Pamela Buffett Cancer Center, Omaha, NE 68198 USA; 3 Omaha Western Iowa Health Care System, VA Service, Department of Research Service, Omaha, NE 68105 USA; 4 Department of Internal Medicine, University of Nebraska Medical Center, Omaha, NE 68105 USA; 5 Division of Urologic Surgery, Department of Surgery, University of Nebraska Medical Center, Omaha, NE 68198 USA; 6 School of Basic and Applied Sciences, Dayananda Sagar University, Bangalore, Karnataka 560078 India; 7 Department of Pathological Physiology, Medical Academy named after S. I. Georgievsky, V. I. Vernadsky Crimean Federal University, Simferopol, Russia 295051; 8 Laboratory of Molecular Biology, Medical Academy named after S. I. Georgievsky, V. I. Vernadsky Crimean Federal University, Simferopol, Russia 295051; 9 Saint Luc’s Clinique, V. I. Vernadsky Crimean Federal University, Simferopol, Russia 295051; 10 Center for Biotechnology, University of Nebraska-Lincoln, Lincoln, NE 68588 USA; 11 Department of Statistics, University of Nebraska-Lincoln, Lincoln, NE 68588 USA; 12 The Nebraska Center for Integrated Biomolecular Communication, University of Nebraska-Lincoln, Lincoln, NE 68588 USA; 13 Department of Chemistry, University of Nebraska-Lincoln, Lincoln, NE 68588 USA; 14 Nanoimaging Core Facility, University of Nebraska Medical Center, Omaha, NE 68105 USA

**Keywords:** Prostate cancer, ER stress, Alcohol abuse, Golgi fragmentation

## Abstract

**Background:**

The development of persistent endoplasmic reticulum (ER) stress is one of the cornerstones of prostate carcinogenesis; however, the mechanism is missing. Also, alcohol is a physiological ER stress inducer, and the link between alcoholism and progression of prostate cancer (PCa) is well documented but not well characterized. According to the canonical model, the mediator of ER stress, ATF6, is cleaved sequentially in the Golgi by S1P and S2P proteases; thereafter, the genes responsible for unfolded protein response (UPR) undergo transactivation.

**Methods:**

Cell lines used were non-malignant prostate epithelial RWPE-1 cells, androgen-responsive LNCaP, and 22RV1 cells, as well as androgen-refractory PC-3 cells. We also utilized PCa tissue sections from patients with different Gleason scores and alcohol consumption backgrounds. Several sophisticated approaches were employed, including Structured illumination superresolution microscopy, Proximity ligation assay, Atomic force microscopy, and Nuclear magnetic resonance spectroscopy.

**Results:**

Herein, we identified the *trans*-Golgi matrix dimeric protein GCC185 as a Golgi retention partner for both S1P and S2P, and in cells lacking GCC185, these enzymes lose intra-Golgi situation. Progression of prostate cancer (PCa) is associated with overproduction of S1P and S2P but monomerization of GCC185 and its downregulation. Utilizing different ER stress models, including ethanol administration, we found that PCa cells employ an elegant mechanism that auto-activates ER stress by fragmentation of Golgi, translocation of S1P and S2P from Golgi to ER, followed by intra-ER cleavage of ATF6, accelerated UPR, and cell proliferation. The segregation of S1P and S2P from Golgi and activation of ATF6 are positively correlated with androgen receptor signaling, different disease stages, and alcohol consumption. Finally, depletion of ATF6 significantly retarded the growth of xenograft prostate tumors and blocks production of pro-metastatic metabolites.

**Conclusions:**

We found that progression of PCa associates with translocation of S1P and S2P proteases to the ER and subsequent ATF6 cleavage. This obviates the need for ATF6 transport to the Golgi and enhances UPR and cell proliferation. Thus, we provide the novel mechanistic model of ATF6 activation and ER stress implication in the progression of PCa, suggesting ATF6 is a novel promising target for prostate cancer therapy.

**Supplementary Information:**

The online version contains supplementary material available at 10.1186/s13046-021-02066-7.

## Background

The link of intracellular stresses to tumorigenesis and tumor growth promotion has been the subject of a decades-long debate. The environment of the endoplasmic reticulum (ER) undergoes significant modifications in response to neoplastic transformations, including oxidative stress, DNA damage, aerobic glycolysis, and calcium deprivation [1–3]. These insults trigger ER stress, a condition under which unfolded/misfolded proteins accumulate within the ER and launch the unfolded protein response (UPR) [4]. It is becoming clear that ER stress is directly linked to the maintenance of the metabolic homeostasis of cancer cells and the adjustment of their microenvironment for tumor survival and expansion [5]. Moreover, ER stress and UPR play a crucial role in the production of antiapoptotic factors and pro-inflammatory cytokines, proliferation, and angiogenesis [6–8]. Therefore, cancer cells utilize a sophisticated ER stress-mediated pathway to sustain proliferative signaling and survival, which is yet to be fully understood.

Activating Transcription Factor 6 (ATF6) governs one important UPR branch. ATF6 is a type II transmembrane protein of the ER. In response to ER stress, 90 kDa ATF6 (p90) dissociates from GRP78/BiP and translocates to the Golgi to be cleaved in its luminal domain by site-1 protease (S1P). Afterward, the N-terminus of ATF6 is cleaved by site-2 protease (S2P), releasing the 50 kDa cytosolic domain (p50). The p50 ATF6 moves to the nucleus, where it initiates transcription of the genes involved in the resistance of ER stress and UPR [9]. Activation of ATF6, as well as the other two branches of UPR, IRE1α and PERK, have been shown in a wide range of solid and hematopoietic malignancies [10]. Several recent studies have implicated ER stress and UPR in the development of prostate cancer (PCa) and the progression of castration-resistant prostate cancer (CRPC) [11, 12]. Chronic ER stress and enhanced activity of ATF6 in PCa are well documented [11–14], but the mechanism is currently unknown.

Recent investigations indicate that Golgi disorganization is a hallmark of cancer progression [15–18]. Additionally, our group recently identified a fragmented Golgi phenotype in PCa cells, which correlated with the progression of this disease [19]. In a recent publication, we introduced the concept of an “onco-Golgi,” which postulates that Golgi disorganization is associated with the activation of various pro-oncogenic and pro-metastatic pathways triggered by the mislocalization of resident Golgi enzymes [20, 21]. Given that ATF6 signaling is the only Golgi-related response among the three UPR pathways, we hypothesized that advanced prostate tumor cells, which display a fragmented Golgi, may utilize a self-activating mechanism of sublethal ER stress.

Using different models of ER stress in various PCa cell lines and analyzing hundreds of prostate tumor samples, we discovered that Golgi proteases S1P and S2P can stay and function in the ER, thereby facilitating the cleavage of ATF6 and amplifying UPR. Additionally, we demonstrated that this mechanism is particularly important in the context of how alcohol affects the progression of PCa.

## Methods

### In vivo mice xenograft model

Male athymic nude mice (BALB/c nu/nu, 20–22 g, 5–6 weeks old; Jackson Laboratory) were individually housed in filter-top cages at the University of Nebraska Medical Center (UNMC) animal facility and consumed food and fresh tap water ad libitum. Food consumption and body weights were recorded weekly. The animals received alcohol orally in drinking water. The amount of alcohol was increased gradually from 4 to 14% within one week. Control mice received water with the appropriate isocaloric amount of sucrose. On the first day of 14% EtOH administration, both groups were inoculated with LNCaP (c-28) cells by subcutaneous injection in the flank 5 × 10^6^ cells in 50 μl of plain RPMI medium plus 50 μl Matrigel (Corning). In another series of xenograft models, mice were injected with LNCaP cells transfected with control or ATF6 shRNA. Tumor diameter was measured weekly throughout the study. This protocol was approved by the Institutional Animal Care and Use Committee (IACUC) at UNMC.

### Patient-derived tissue samples and lysates

Tissue sections from the normal prostate were obtained from US Biomax and Novus Biological. Also, sections were provided through the Department of Pathology and Microbiology (IRB protocol # 304–16-EP) at the University of Nebraska Medical Center, the Johns Hopkins University School of Medicine (Prostate Cancer Biorepository Network), and the Vernadsky Crimean Federal University (Russia). Patient questionnaires asked for a) preferred alcoholic beverage(s); b) an average number of drinks consumed in a week within the last five years; c) frequency of heavy episodic drinking and d) duration of heavy drinking occasions. We monitored the PLA signal in the pairs of proteins, S1P/GCC185 and S2P/GCC185, in normal prostate and PCa cells from patients with the same grade and Gleason score who are non-drinking patients (who do not drink or drink less than once per month) versus patients who regularly consume alcohol at a moderate level (12 oz. beer – 5-6 times per week; 3–5 glasses of wine per week; 3.4 oz. of strong liquor – 2-3 times per week) or at a heavy level (12 oz. beer – 2 or more times per day; 4 oz. glass of wine – 1 or more times per day; 3.4 oz. of strong liquor at least once per day). Lysates isolated from normal prostate cells and tumors of PCa patients were obtained from Protein Biotechnologies (USA).

### The details of other methods and materials are described in the supplemental information

These include: Antibodies and reagents; Cell culture and EtOH treatment; Soft agar assay for colony formation; Cell migration assay; Immunohistochemistry; Confocal immunofluorescence microscopy; Three-dimensional structured illumination (3D-SIM) microscopy and image analysis; AFM imaging and image analysis; In situ Proximity Ligation Assay (PLA); NMR data collection and analysis; Isolation of Golgi membrane fractions by sucrose gradient centrifugation; Isolation of microsomal fraction; Immunoprecipitation (IP), plasmid constructions, and transfection; Quantitative gene expression analysis by qRT-PCR; Quantification and statistical analysis.

### Results

### Prostate cancer cells demonstrate a high level of ATF6-mediated ER stress

**Fig. 1:**
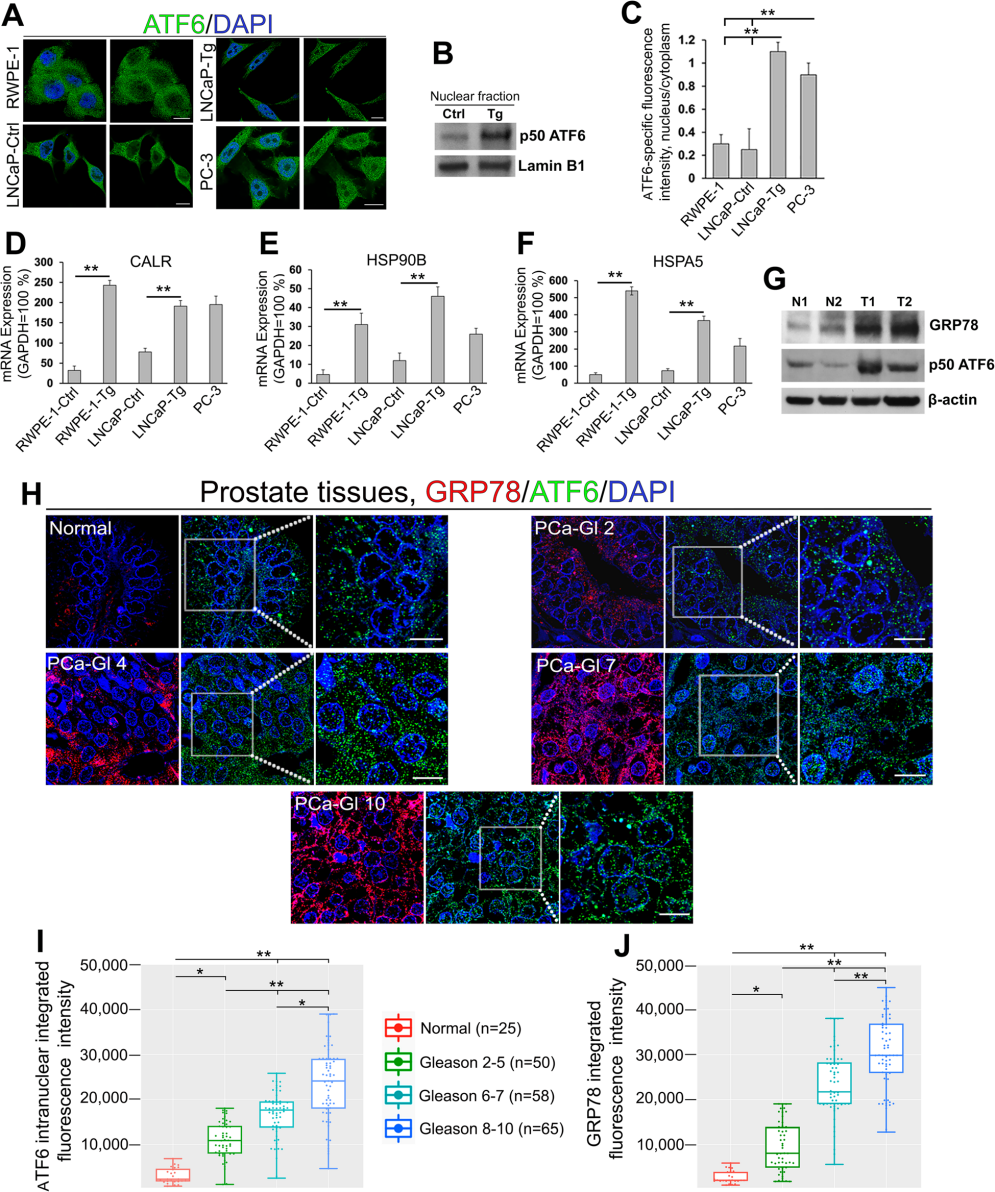
ATF6 mediated ER stress in PCa cells. (**A**) Representative immunofluorescence (IF) images of ATF6 (green) in LNCaP cells treated with 1 μM Thapsigargin (Tg) for 4 h or the appropriate amount of DMSO (Ctrl) and non-treated RWPE-1 and PC-3 cells; bars, 10 μm. (**B**) ATF6 W-B of the nuclear fraction from LNCaP cells treated with DMSO or Tg; lamin B1 is a loading control. (**C**) Quantification of the ratio of nuclear/cytoplasmic ATF6 IF in cells presented in A (*N* = 3; **P < 0.001, *t* test). (**D-F**) Expression of mRNA for calreticulin (**D**), HSP90B (**E**), and HSPA5 (**F**) in RWPE-1 and LNCaP cells treated with DMSO or Tg, as well as non-treated PC-3 cells (N = 3; **P < 0.001, *t* test). (**G**) GRP78 and ATF6 W-B of the lysate from the tumor tissues of PCa patients (T, grade 2) and prostate normal tissues (N); β-actin is a loading control. (**H**) Examination of intranuclear ATF6 and total GRP78 in the normal prostate and tumor foci of PCa patients with different Gleason (Gl) scores. Shown is IF staining of GRP78 (red) and ATF6 (green); bars, 5 μm. White boxes indicate an area enlarged at the right. (I, J) Quantification of ATF6 (**I**) and GRP78 (**J**) in the samples described in H (**P < 0.001; *P < 0.01, pairwise Wilcoxon with Bonferonni-Hochberg multiple test). Data are presented as medians (min – max)

To characterize the level of ATF6-mediated ER stress, we measured the expression of cleaved ATF6 in non-malignant prostate epithelial RWPE-1 cells, androgen-responsive LNCaP cells c-24 (hereafter LNCaP cells), and androgen-refractory PC-3 cells. As shown in Fig. 1A and B, in RWPE-1 and LNCaP cells, only a marginal expression of nuclear ATF6 was detected, whereas treatment of LNCaP cells with the ER stress inducer Thapsigargin (Tg, 1 μM for 4 h) resulted in aggregation of cleaved ATF6 within the nucleus. In the meantime, a robust ATF6 signal can be detected in the nuclei of non-treated PC-3 cells. Quantification of ATF6-specific immunofluorescence in the nucleus vs. the cytoplasm implied that highly aggressive PC-3 cells are in a state of constant stress comparable to Tg-treated low aggressive LNCaP cells (Fig. 1C). Cleaved ATF6-inducible genes include GRP78/BiP, also known as HSPA5, calreticulin (CALR), and HSP90b, also known as GRP94. We compared the basal expression of these genes in non-treated PC-3 cells to that of Tg-treated RWPE-1 and LNCaP cells. As shown in Fig. 1D-F, the CALR gene expression in both RWPE-1 and LNCaP cells increased significantly after Tg treatment and approximated to that in PC-3 cells. The baseline level of HSP90B and HSPA5 mRNA in PC-3 cells was significantly higher than in the untreated RWPE-1 and LNCaP cells; however, expression of these genes in Tg-treated RWPE-1 and LNCaP cells were higher than in PC-3. Next, we found that the expression of GRP78 and cleaved ATF6 was enhanced in the lysate of PCa cells isolated from patient tumors compared to samples isolated from normal prostate (Fig. 1G). Based on these observations, we proceeded to test whether the expression of GRP78 and nuclear ATF6 correlate with the aggressiveness of PCa. For both proteins, a significant difference between normal prostate and PCa was immediately detectable in patients with Gleason scores 2–5 (Fig. 1H-J). Furthermore, when combining Gleason scores 2–5, 6–7, and 8–10, we observed that both parameters were reliably higher and significantly distinct between these groups of PCa patients (*P* < 0.001, *P* < 0.01, pairwise Wilcoxon with Bonferonni-Hochberg multiple test). Overall, the data presented in Fig. 1 imply an intrinsic mechanism used by PCa cells to activate the ATF6 branch of ER stress, which is positively correlated with the increasing rate of Gleason scores.

### Golgi localization of both S1P and S2P proteases requires GCC185

**Fig. 2:**
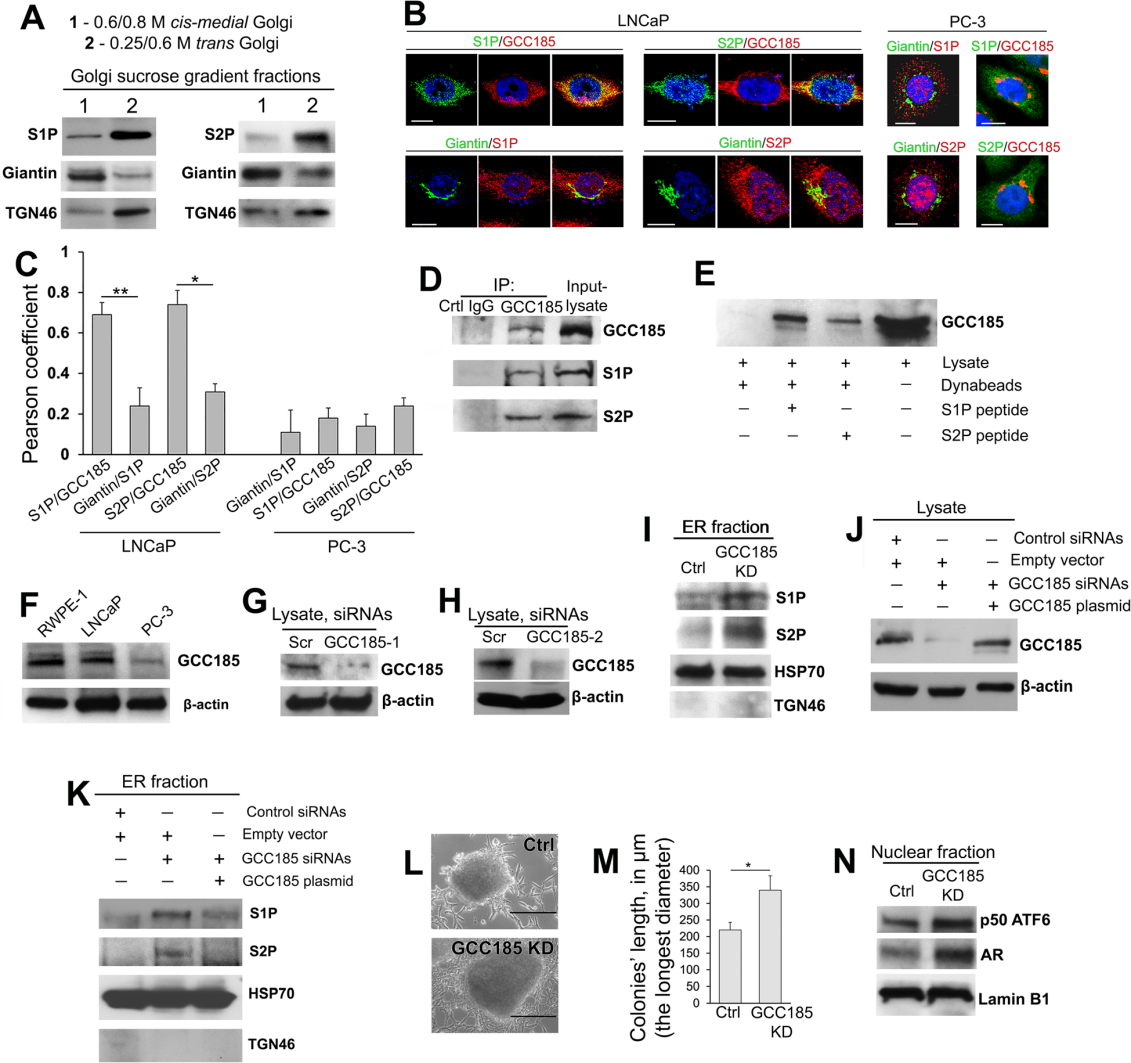
GCC185 is the Golgi binding partner for S1P and S2P. (**A**) S1P and S2P W-B of the *cis-medial*-Golgi and *trans*-Golgi fractions (0.6 M/0.8 M and 0.25 M/0.6 M sucrose interface, respectively); samples were normalized by the total protein concentration. Giantin and TGN46 were used as loading controls for *cis-medial-* and *trans*-Golgi, respectively. (**B**) IF staining of LNCaP cells (left panel) and PC-3 cells (right panel) to estimate colocalization of S1P and S2P with giantin and GCC185; bars, 10 μm. (**C**) Quantification of the Pearson coefficient of colocalization for the cells presented in B (*N* = 90 cells from three repeats; **P < 0.001, *P < 0.01, *t* test). (**D**) S1P and S2P W-B of the GCC185 immunoprecipitation (IP) sample prepared from LNCaP cells. (**E**) GCC185 W-B of the lysate or the protein complex pulled down from the lysate of LNCaP cells using biotinylated hS1P or hS2P full-length peptides and Dynabeads M-280 Streptavidin. (**F**) GCC185 W-B of the lysate of RWPE-1, LNCaP, and PC-3 cells; β-actin is a loading control. (**G, H**) GCC185 W-B of the lysate of LNCaP cells treated with scramble or different combinations of GCC185 siRNAs. (**I**) S1P and S2P W-B of the ER fraction isolated from LNCaP cells: control and GCC185 KD; HSP70 and TGN46 are loading controls for ER and *trans*-Golgi, respectively. (**J**) GCC185 W-B of the lysates from LNCaP cells: transfected with control siRNAs and empty pCMV6-AC vector, transfected with GCC185 siRNAs and empty pCMV6-AC vector, and transfected with GCC185 siRNAs followed by GCC185 plasmid corresponding to the WT hGCC185. (**K**) S1P and S2P W-B of the ER fraction isolated from LNCaP cells presented in J. (**L**) Representative images of colonies formed by control and GCC185 KD LNCaP cells as described in the Materials and Methods section; bars, 200 μm. (**M**) Quantification of the colonies’ lengths (the longest diameter) in 30 randomly selected areas for control and GCC185 KD LNCaP cells. Data were collected from three independent experiments and expressed as a mean ± SD; * *P* < 0.01, *t* test. (**N**) ATF6 and AR W-B of the nuclear fraction from control and GCC185 KD LNCaP cells; lamin B1 is a loading control

It has been suggested that in HeLa cells, S1P and S2P proteases reside within the proximal Golgi compartments [22, 23]; however, their precise localization was not determined. To examine the intra-Golgi distribution of S1P and S2P, we conducted a sucrose gradient (0.25 M/0.6 M/0.8 M) ultracentrifugation of the Golgi membranes isolated from RWPE-1 cells. Western Blots (W-Bs) of these samples (normalized by a total protein concentration) revealed a large portion of S1P and S2P within the *trans*-Golgi fraction; in contrast, only marginal amounts of these proteases were detected in the *cis-medial*-Golgi membranes (Fig. 2A). Next, we proceeded to identify the key *trans*-Golgi matrix protein that could potentially serve as a Golgi tethering factor for both S1P and S2P. The well-studied *trans*-golgins are GCC185, GCC88, TMF, and Golgin-245. First, we examined their colocalization with S1P and S2P and detected a high Pearson coefficient between S1P and GCC185 (~ 0.76) and then S2P and GCC185 (~ 0.72) (Fig. S1A-C). In the meantime, the level of IF overlay between GCC88, TMF, or Golgin-245 and S1P or S2P was negligible and significantly lower than that in pairs S1P/GCC185 and S2P/GCC185 (*P* < 0.001 and < 0.01, *t* test) (Fig. S1A-C). To validate these results, we performed an S1P and S2P immunoprecipitation (IP) followed by a W-B with each of these Golgi proteins. We found no direct physical co-association of S1P or S2P with GCC88, TMF, and Golgin-245; however, a fraction of GCC185 was detectable in both S1P and S2P IP samples, suggesting a complex formed between these proteases and GCC185 (Fig. S1D). To evaluate the applicability of the results obtained from RWPE-1 cells to PCa cell lines, we measured in LNCaP and PC-3 cells the colocalization of S1P and S2P with giantin (*cis*-*medial*-Golgi) and GCC185 (*trans*-Golgi). In LNCaP cells, the Pearson coefficient in pairs S1P/GCC185 and S2P/GCC185 was significantly higher than that in S1P/giantin and S2P/giantin, respectively (*P* < 0.001 and < 0.01, *t* test) (Fig. 2B and C, left panel), confirming the predominant distribution of both proteases in the *trans*-Golgi. Conversely, PC-3 cells demonstrated segregation of both S1P and S2P from either giantin or GCC185 (Fig. 2B and C, right panel), indicating that these enzymes were distributed outside of Golgi. Notably, PC-3 cells exhibit fragmented Golgi, but RWPE-1 and LNCaP cells have a compact and perinuclear Golgi [19, 21]. Therefore, it appears that Golgi scattering is associated with the loss of Golgi residency for both S1P and S2P.

Next, using LNCaP cells, we validated the complexes S1P↔GCC185 and S2P↔GCC185 by detecting both S1P and S2P in the GCC185 IP sample (Fig. 2D). Further, a direct interaction between these proteins was evaluated with the following procedure. The biotinylated peptides corresponding to the full-length hS1P and hS2P were immobilized using magnetic Dynabeads then incubated with LNCaP cell lysate. As shown in Fig. 2E, Dynabeads carrying either the S1P or S2P peptides could pull down GCC185; however, GCC185 was not detected in the pull-down fraction from the lysate, which was only exposed to the Dynabeads. In addition, we observed a substantial decline in the expression of GCC185 in PC-3 cells compared to RWPE-1 and LNCaP cells (Fig. 2F).

### The fragmented Golgi phenotype of PCa cells is associated with monomerization of GCC185 and the translocation of S1P and S2P from Golgi to the ER

These results, in addition to the lack of S1P and S2P in the Golgi of PC-3 cells, led us to conclude that GCC185 is critical for the Golgi localization of these proteases, and downregulation of GCC185 could be a cause for the translocation of S1P and S2P from the Golgi to the ER. We performed a siRNA-induced knockdown (KD) of GCC185 in LNCaP cells (Fig. 2G and H) and measured by W-B the content of S1P and S2P within the ER fraction. A series of repeat experiments from different GCC185 siRNAs clearly showed a robust increase of both proteases in the ER from the cells lacking GCC185 (Fig. 2I). The rescue experiment was performed using overexpression of exogenous GCC185. As shown in Fig. 2J, cells transfected with GCC185 siRNAs and empty pCMV6-AC vector demonstrate KD of GCC185. However, when cells were transfected with GCC185 siRNAs followed by GCC185 plasmid corresponding to the WT hGCC185, the expression of GCC185 was closed to that in cells transfected with control siRNAs and empty pCMV6-AC vector. Importantly, restoration of GCC185 level preserves S1P/S2P shift to the ER (Fig. 2K), confirming the negative link between expression of GCC185 and level of S1P/S2P in the ER. Next, we detected that GCC185 KD cells demonstrate a larger size of colonies compared with control cells, indicating a higher rate of proliferation (Fig. 2L and M). Also, in the nuclear fraction of cells lacking GCC185, we detected the increased content of both cleaved ATF6 and AR (Fig. 2N), which clearly shows that ATF6-mediated ER stress and AR transactivation can be ascribed to downregulation of GCC185.

**Fig. 3:**
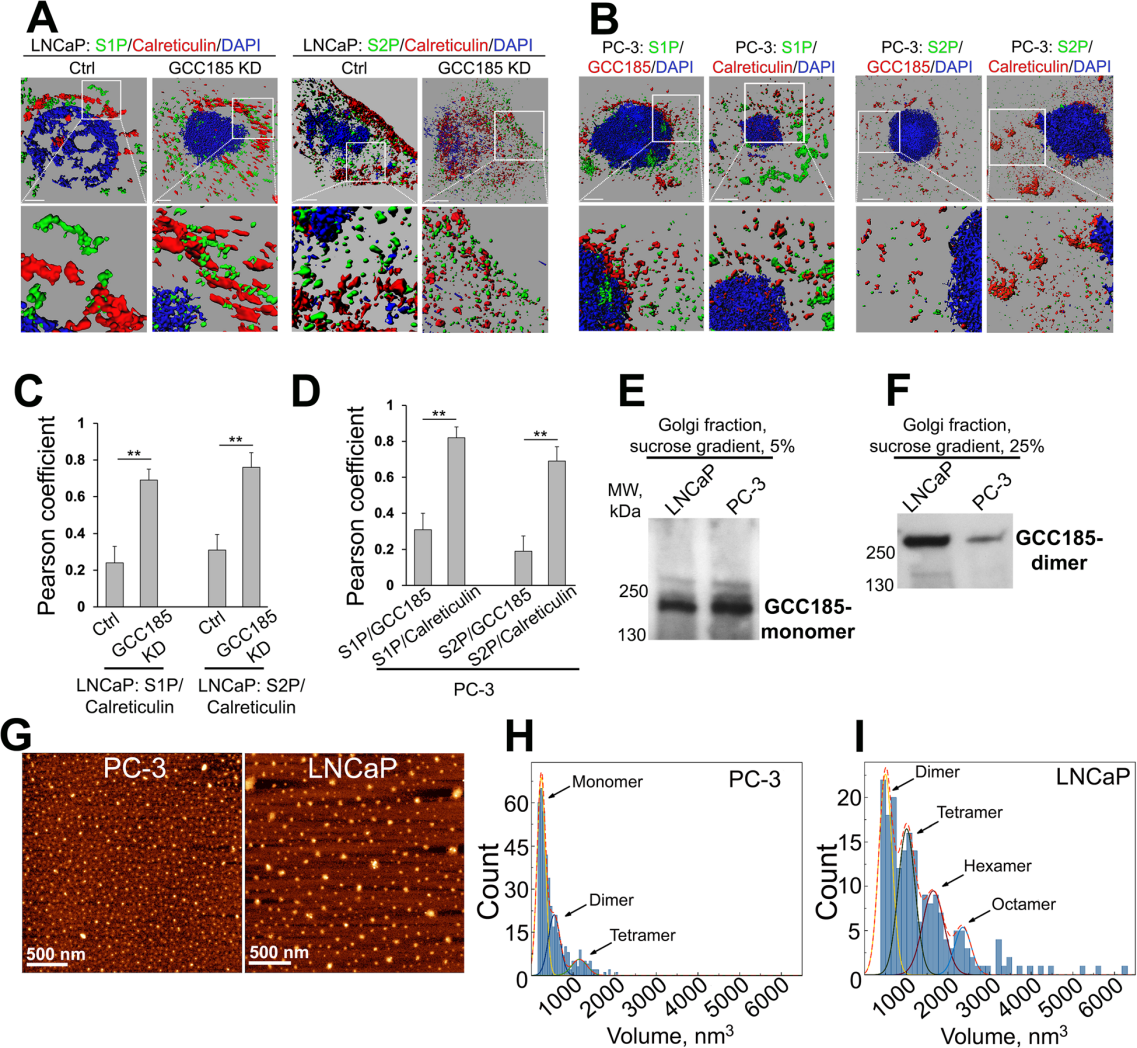
The shift of S1P and S2P to ER is associated with the monomerization of GCC185. (**A**) Representative 3D SIM imaging of LNCaP cells: control and GCC185 KD. Cells were co-stained with S1P (green, left panel) or S2P (green, right panel) and calreticulin (red); bars, 2 μm. White boxes indicate the area magnified below. (**B**) Representative 3D SIM imaging of PC-3 cells co-stained with S1P or S2P (green) and GCC185 or calreticulin (red); bars, 2 μm. (**C, D**) Quantification of the Pearson coefficient of colocalization for the indicated proteins in cells from A and B, respectively (*N* = 10 cells for each series of SD SIM imaging; **P < 0.01, *t* test). (**E, F**) Golgi fractions isolated from LNCaP and PC-3 cells were subjected to sucrose sedimentation analysis on a 5–25% sucrose gradient. 5% (**E**) and 25% (**F**) fractions were collected and analyzed by 4–15% gradient SDS-PAGE and probed with GCC185 Ab. The samples were prepared under low (1%) concentrations of β-mercaptoethanol, and the same amount of proteins were loaded. (**G**) AFM topography images of GCC185 from PC-3 and LNCaP cells; bar, 500 nm. (**H** and **I**) Statistical histogram of GCC185 protein volume distribution for PC-3 (H, *n* = 424) and LNCaP (I, *n* = 194) samples

We further verified the segregation of S1P and S2P from Golgi by structured illumination superresolution microscopy (SIM), which allows to create 3D reconstructed images with a lateral resolution of ~ 110 nm, approximately twice that of diffraction-limited instruments. First, in LNCaP cells, we measured colocalization of S1P and S2P with the ER marker calreticulin before and after GCC185 KD. Contrary to the control, cells with depleted GCC185 demonstrated a high level of colocalization between both proteases and calreticulin (*P* < 0.01, *t* test) (Fig. 3A and C). Second, we proceeded to examine the colocalization of S1P and S2P with GCC185 and calreticulin in PC-3 cells. The calculated Pearson coefficient of colocalization confirmed that S1P and S2P are predominantly distributed within the ER but not in the fragmented Golgi membranes (*P* < 0.01, *t* test) (Fig. 3B and D), echoing the results of the IF analysis presented in Fig. 2B and C. Interestingly, the same phenomenon was observed in RWPE-1 and LNCaP cells experiencing ER stress after Tg treatment. Indeed, the number of cells with fragmented Golgi and the expression of GRP78 was robustly increased after Tg treatment and was comparable to that of the non-treated PC-3 cells (*P* < 0.001, *t* test) (Fig. S2A-C). Finally, in LNCaP cells, we found that Tg treatment increased S1P and S2P in the ER fractions but reduced their content in the Golgi membranes (Fig. S2D and E). In sum, these results indicate that GCC185 is required for Golgi localization of S1P and S2P, and ER stress is associated with Golgi disorganization and the translocation/retention of these proteases to the ER. This concept fits well with observations of others that upon ER stress induced by Golgi-disrupting agent Brefeldin A, S1P and S2P relocate to the ER, where they can cleave ATF6 [22, 23].

Recent work from our lab and others suggests that in ER stressed cells, Golgi disorganization is associated with the monomerization of the Golgi scaffold proteins, including giantin and GRASP55 [24, 25]. Moreover, we observed that in LNCaP cells, giantin was present primarily in its dimeric form, whereas in PC-3 cells, giantin remained largely monomeric [19]. Consequently, several giantin-dependent Golgi enzymes translocate to the ER, which undoubtedly affects glycosylation, resulting in the formation of pro-metastatic glycan profiles [19, 26]. This prompted us to analyze whether the same phenomenon occurs to the GCC185 protein. Using sucrose gradient (5–25%) sedimentation analysis of the Golgi membranes, we detected monomeric GCC185 in the light 5% fraction of both LNCaP and PC-3 cells (Fig. 3E). However, contrary to the LNCaP cells, the content of GCC185 dimer in the heavy 25% fraction of PC-3 cells was much less (Fig. 3F). To visualize GCC185 dimerization, we performed Atomic Force Microscopy (AFM) imaging. We isolated endogenous GCC185 from both LNCaP and PC-3 cells using the IP method described previously by our group [24]. Under such conditions of isolation, proteins are folded into stable globular structures, clearly identified in the images as bright spots. Figure 3G shows representative AFM topography images of GCC185 proteins from LNCaP and PC-3 cells after deposition on the APS substrate. Using a “spherical cap” model [24], we estimated the volume of proteins. In PC-3 cells, we detected two peaks with maxima at 237 ± 5 nm^3^ and 547 ± 15 nm^3^, corresponding to the monomeric and dimeric GCC185, respectively (Fig. 3H). In the meantime, the first peak from LNCaP cells was detected with a maxima 502 ± 16 nm^3^, implying the predominant dimeric form of GCC185 (Fig. 3I). Intriguingly, in PC-3 cells, we observed a small “shoulder” with a volume of 1152 ± 56 nm^3^, corresponding to GCC185 tetramer. This, however, was detected as a strong peak in the LNCaP sample, with a maxima 1010 ± 21 nm^3^. Moreover, GCC185 from LNCaP samples demonstrated additional peaks with volumes of 1650 ± 37 nm^3^ and 2359 ± 51 nm^3^, corresponding to GCC185 hexamers and octamers, respectively (Fig. 3H and I).

### Examination of S1P/S2P and GCC185 closeness in prostate tumor samples

**Fig. 4:**
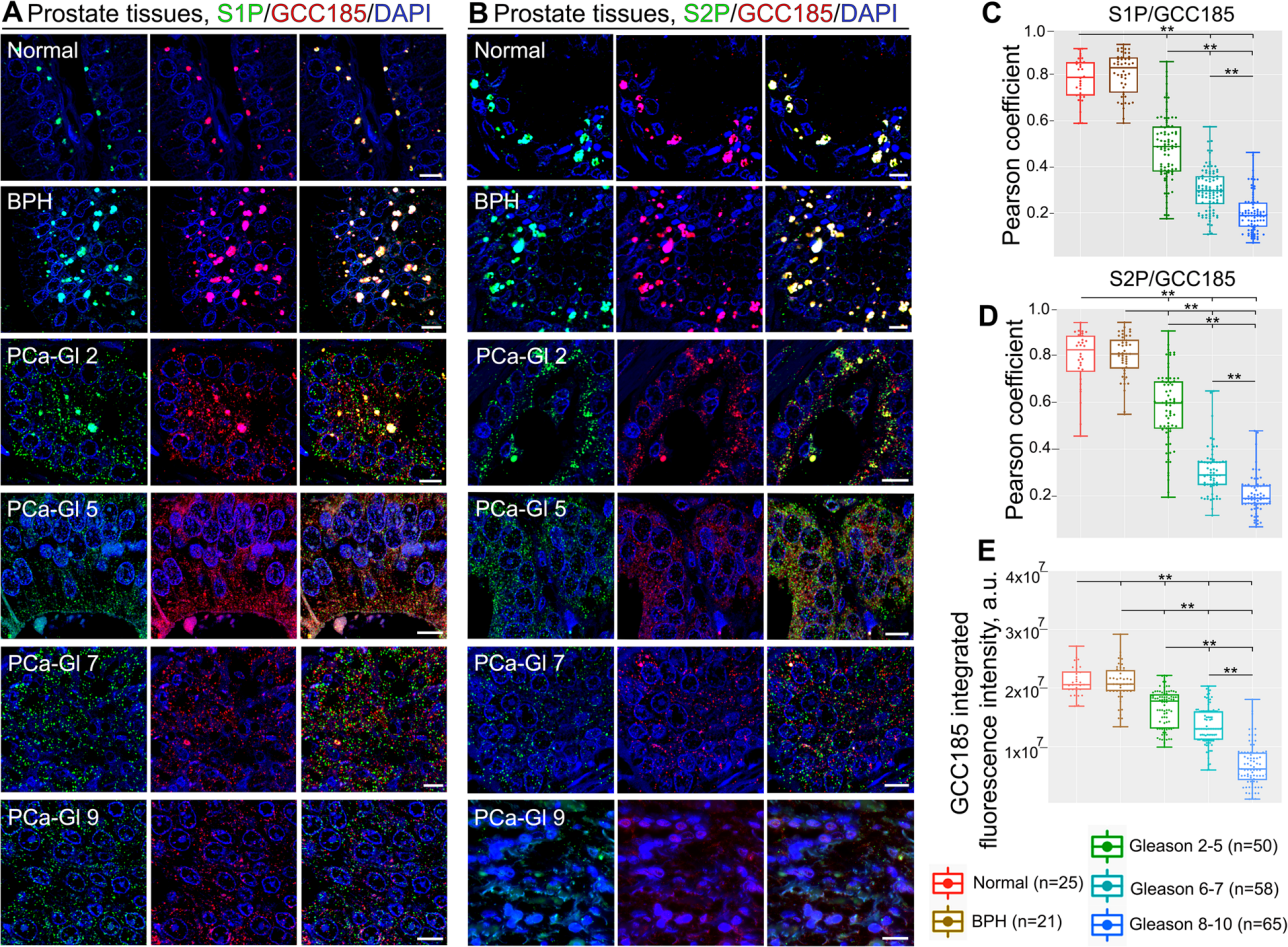
Colocalization of GCC185 with S1P and S2P in the prostate tissues. (**A** and **B**) IF of S1P (green)/GCC185 (red) (**A**) and S2P (green)/GCC185 (red) (**B**) in representative normal prostate tissues, BPH, and PCa tissues with different Gleason scores. All confocal images were acquired with the same imaging parameters; bars, 5 μm. (**C** and **D**) Quantification of Pearson coefficient of colocalization in pairs S1P/GCC185 (**C**) and S2P/GCC185 (**D**) in the samples described in A and B, respectively (**P < 0.001 between normal, BPH and each group of PCa, as well as between every group of PCa, pairwise Wilcoxon with Bonferonni-Hochberg multiple test). No significant difference was found between normal prostate and BPH. The threshold for GCC185 was normalized for all samples, given the reduction of signal in PCa tissues. (**E**) Quantification of GCC185 integrated fluorescence intensity (in a.u.) in the representative normal prostate tissues, BPH, and PCa tissues with different Gleason scores presented in A and B. Data are presented as medians (min – max); (**P < 0.001, *P < 0.05, *t* test)

In sum here, these results indicate that in cells that have a compact Golgi, GCC185 may form oligomeric complexes and serve as a Golgi retention partner for S1P and S2P. ER stress associates with Golgi disorganization, monomerization of GCC185, and the ER residency for S1P and S2P. To examine the clinical applicability of these findings, we measured the colocalization of both proteases with GCC185 in PCa tissue samples. The colocalization rate in the S1P/GCC185 and S2P/GCC185 pairs was high and almost identical in the normal prostate tissue and tumor samples from patients with benign prostatic hyperplasia (BPH) (Fig. 4A-D). However, the Pearson coefficient was significantly lower in the groups of PCa patients with Gleason scores 2–5, 6–7, and 8–10. Notably, a powerful difference was detected between these groups, implying a correlation between S1P and S2P segregation from the Golgi and the severity of this pathology (Fig. 4A-D). Statistical analysis of GCC185 IF revealed no difference in its expression between normal prostate tissue and BPH. By contrast, the IF signal of GCC185 was declined in PCa tissues (*P* < 0.001, *t* test), which was correlated with Gleason scores, according to a significant difference found between Gleason score groups 2–5 (*P* < 0.05, *t* test), 6–7, and 8–10 (*P* < 0.001, *t* test) (Fig. 4E). Then, we estimated the protein level of S1P and S2P by immunohistochemistry. Analogously, we could not find a difference between the normal prostate tissue adjacent to the tumor (NAT) and BPH. However, in the PCa group, both S1P and S2P demonstrate a strong positive signal, significantly higher than in NAT and BPH (Fig. S3).

A quantitative in situ proximity ligation assay (PLA) was carried out in PCa samples to corroborate these data by an alternative sensitive technique. PLA can visualize protein-protein interaction by the red fluorescence emanating from the proximity (below 40 nm) of these two proteins. Here, we measured the PLA-specific red fluorescent spots to detect the proximity of S1P and S2P to GCC185. In addition, we sought to assess whether the level of segregation of these proteases from the Golgi correlates with the aggressive potential of PCa. The technical details of the experiments and our modification for the image processing are described in Fig. S4A. We examined tissue samples from two groups of PCa patients: (a) with Gleason scores 6 and 7, and (b) Gleason scores from 8 to 10. In all tissue samples, PLA signals from both pairs S1P/GCC185 and S2P/GCC185 were markedly declined when compared to the normal prostate (*P* < 0.001, Kruskal-Wallis omnibus test) (Fig. S4B-E). It was also found that in the group of patients with Gleason scores 6 and 7, S1P/GCC185-specific PLA signal was significantly higher than that in tumors representing Gleason scores 8, 9, and 10 (Fig. S4B-E) (*P* < 0.001, pairwise Wilcoxon with Bonferonni-Hochberg multiple test). Consistent with the data presented in Fig. 1H-J, these results indicate a strong association between S1P and S2P intracellular translocation, ATF6-mediated ER stress and the progression of PCa.

### The expression of ATF6 correlates with intranuclear AR in different clinical stages of prostate tumors and PCa cell lines. Depletion of ATF6 blocks xenograft tumor growth

Androgens and androgen receptor (AR) signaling pathways are commonly considered the main oncogenic drivers of prostate carcinogenesis [27]. Moreover, AR persists in the majority of patients with CRPC [28]. Here, the correlation between intranuclear ATF6 and AR was rigorously analyzed using clinical samples obtained from normal prostate, NAT, BPH, BPH with Low-Grade Prostatic Intraepithelial Neoplasia (BPH LG PIN), BPH with High-Grade Prostatic Intraepithelial Neoplasia (BPH HG PIN), and PCa tissues (Fig. S5A-F). As the variables in all sample groups showed a deviation from normality (*P* < 0.001, D’Agostino-Pearson Omnibus Normality Test), the Spearman correlation coefficient *r*_*s*_ was calculated to determine the ATF6-AR relationship. In all groups, we detected a strong positive correlation between these two parameters; namely, *r*_*s*_ was 0.71 for normal tissue, 0.48 for NAT, 0.64 for BPH, 0.59 for BPH LG PIN, 0.63 for BPH HG PIN, and 0.59 for PCa samples (*P* < 0.001 for every group) (Fig. S5G). Similarly, a strong correlation between intranuclear ATF6 and AR was found in LNCaP (Fig. S6A and C, *r*_*s*_ = 0.58, *P* < 0.001) and 22Rv1 cells (Fig. S6B and D, *r*_*s*_ = 0.55, *P* < 0.001).

**Fig. 5:**
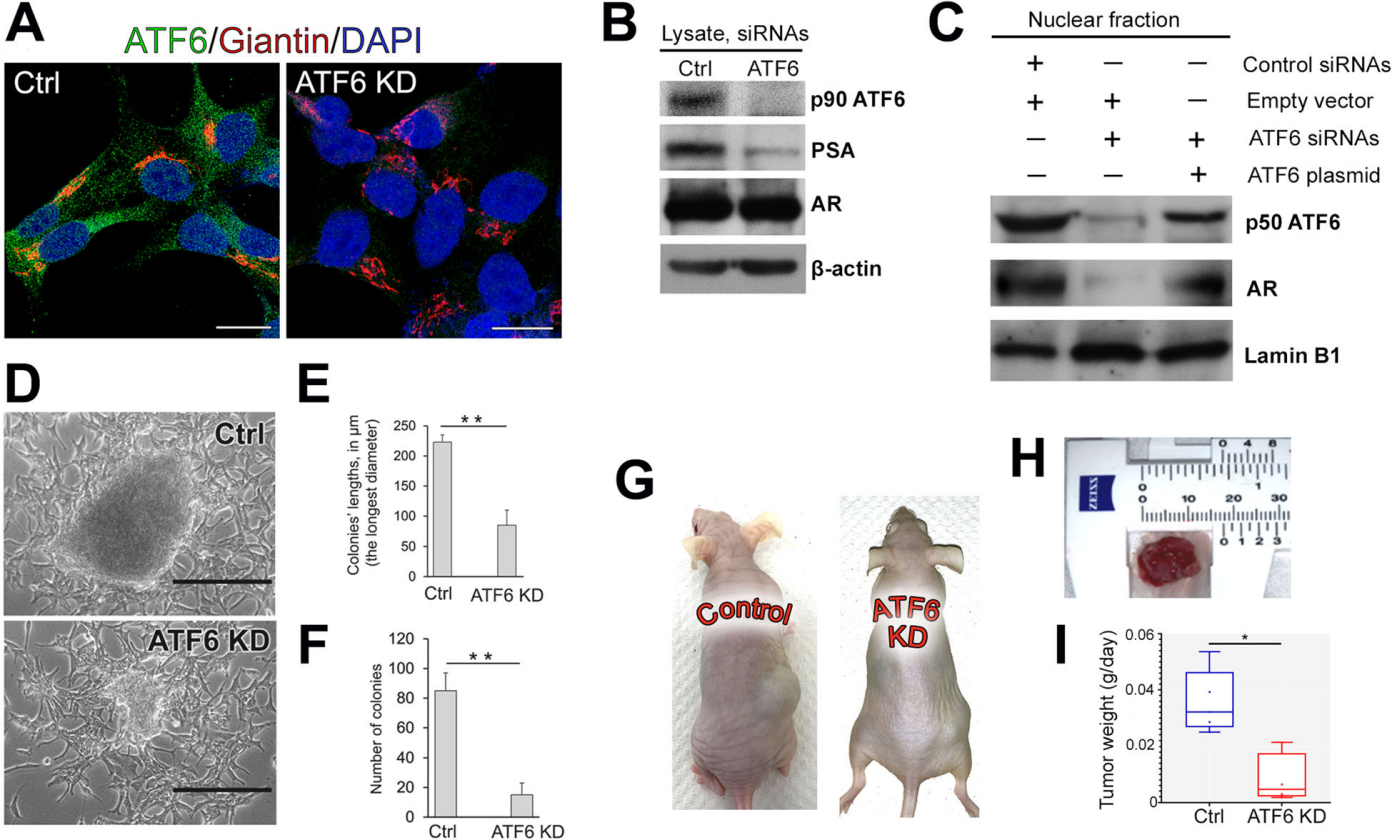
Depletion of ATF6 blocks proliferation of LNCaP cells and growth of xenograft tumors. (**A**) IF staining of ATF6 (green) and giantin (Golgi, red) in LNCaP cells treated with control or ATF6α siRNAs; bars, 10 μm. (**B**) ATF6, PSA, and AR W-B of the lysate of LNCaP cells transfected with control or ATF6 siRNAs; β-actin is a loading control. (**C**) ATF6 and AR W-B of the nuclear fraction from: transfected with control siRNAs and empty pEGFP-N1 vector, transfected with ATF6 siRNAs and empty pEGFP-N1vector, and transfected with ATF6 siRNAs followed by pEGFP-ATF6 plasmid corresponding to the WT hATF6; lamin B1 is a loading control. (**D**) Representative images of colonies formed by control and ATF6 KD LNCaP cells as described in the Materials and Methods section; bars, 200 μm. (**E** and **F**) Quantification of the colonies’ lengths (the longest diameter) (**E**) and the number of colonies (**F**) in 30 randomly selected areas for control and ATF6 KD LNCaP cells. Data were collected from three independent experiments and expressed as a mean ± SD; ** *P* < 0.01, *t* test. (**G**) The xenograft tumors in nude mice inoculated with control and ATF6 KD LNCaP cells. (**H**) Representative tumor derived from Ctrl LNCaP cells. (**I**) The tumor growth is presented as a ratio of tumor weight (g)/number of days after injection. Data were collected from four control and four ATF6 KD xenograft tumors and expressed as medians (min – max); ** *P* < 0.01, *t* test

These data imply that a lack of ATF6 could have a direct impact on AR signaling and subsequent tumor proliferation. We performed siRNA-mediated KD of ATF6 in LNCaP cells, and as shown in Fig. 5A and B, the expression of ATF6 was suppressed. The total level of AR in the cell lysate was also reduced. Importantly, the content of prostate-specific antigen (PSA), a widely used indicator of AR transactivation [29], was significantly lower. Next, in ATF6 KD cells, the level of AR in the nuclear fraction was decreased (Fig. 5C), confirming the tight link between ATF6-mediated ER stress and AR transactivation. Notably, the reintroduction of ATF6 rescued the intranuclear content of both cleaved ATF6 and AR (Fig. 5C).

To assess the proliferation rate and the ability of cells to metastasize, we monitored the anchorage-independent growth by soft agar analysis, as previously described [21]. In ATF6 KD cells, the size and number of colonies were significantly reduced compared to their control counterparts (Fig. 5D-F, *P* < 0.01, *t* test). Next, in LNCaP (C-25-27) cells, we performed the stable KD of ATF6α using shRNA; the tumorigenicity of these cells was assessed following subcutaneous injection into the flank of nude mice (Fig. 5G). Given that some of the tumors were not spherical (Fig. 5H), we decided not to use the widely used formula of tumor volume V = 1/2 (length x width^2). Instead, we counted the ratio of tumor weight/number of days after injection. Quantification showed that cells transfected with control shRNAs demonstrate a high rate of tumor growth compared to the ATF6 KD cells (Fig. 5I, *P* < 0.01, *t* test).

### The validation of ATF6-mediated ER stress in EtOH-treated PCa cells and prostate tumor from alcohol-consuming patients

**Fig. 6:**
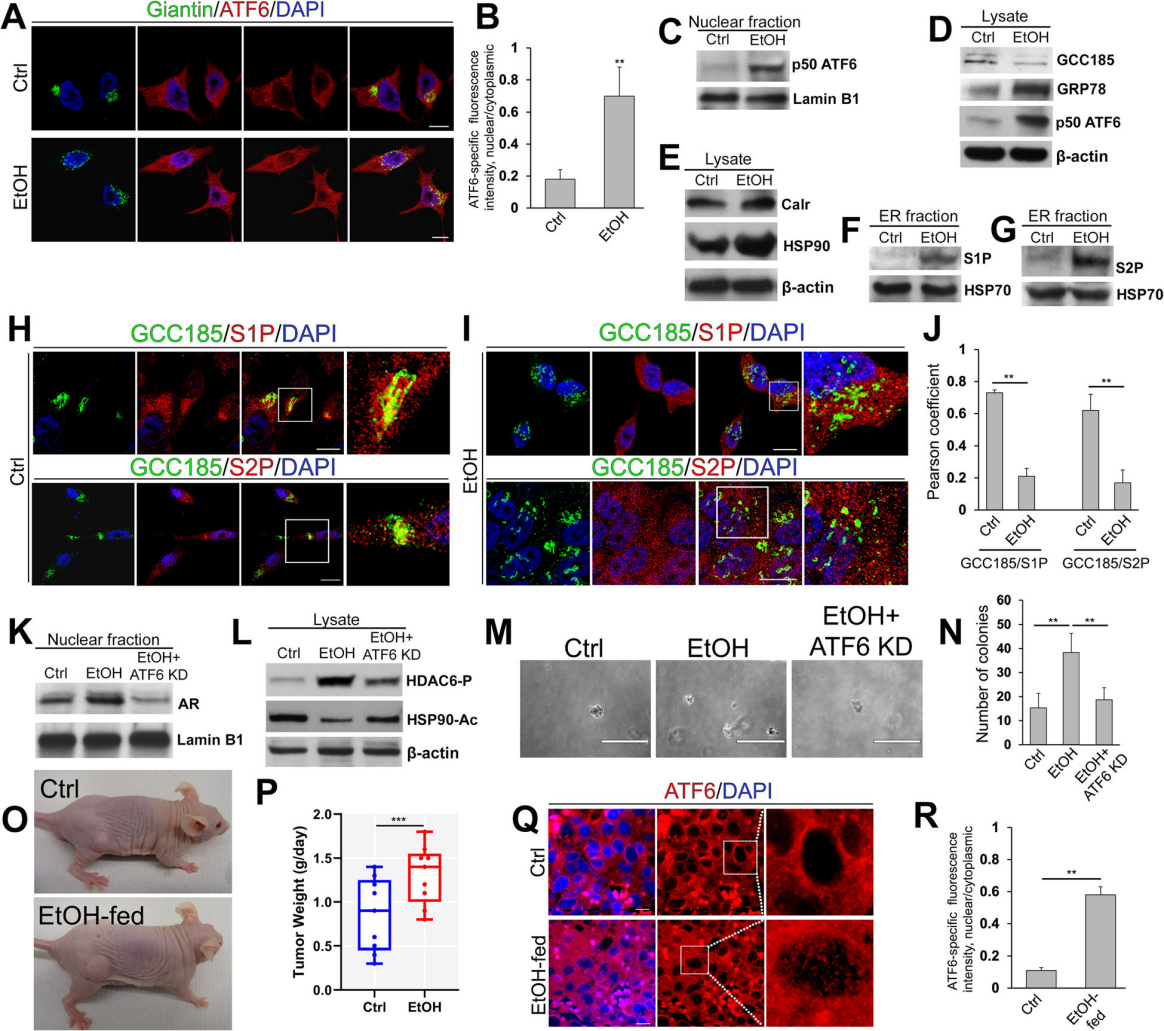
Alcohol-induced ER stress in PCa cells. (**A**) Representative IF images of giantin (green) and ATF6 (red) in LNCaP cells: control and treated with 50 mM EtOH for 96 h; bars, 10 μm. (**B**) Quantification of the ratio of nuclear/cytoplasmic ATF6 IF in cells presented in A (*N* = 3; **P < 0.001, *t* test). (**C**) ATF6 W-B of the nuclear fraction from cells presented in A; lamin B1 is a loading control. (**D**) GCC185, GRP78, and ATF6 W-B of the lysates from the cells presented in A; β-actin is a loading control. (**E**) Calreticulin and HSP90 W-B of the lysates from the cells presented in A; β-actin is a loading control. (**F** and **G**) S1P (**F**) and S2P (**G**) W-B of the ER fraction isolated from the cells presented in A; HSP70 is a loading control. (**H** and **I**) IF staining of LNCaP cells to evaluate colocalization of S1P (red) and S2P (red) with GCC185 (green); bars, 10 μm. White boxes indicate area magnified at the right. (**J**) Quantification of the Pearson coefficient of colocalization for the cells presented in **H** and **I** (*N* = 90 cells from three repeats; **P < 0.001, *t* test). (**K**) AR W-B of the nuclear fraction from LNCaP cells: control, treated with ETOH, and ATF6 siRNA followed by EtOH. (**L**) HDAC6-P and HSP90-Ac W-B of the lysate of cells from K. (**M**) Anchorage-independent colony formation was measured by the soft agar assay for LNCaP cells: control, treated with ETOH, and ATF6 siRNA followed by EtOH; bars, 200 μm. (**N**) Quantification of the number of colonies in 20 randomly selected areas for cells from M (N = 3; **P < 0.001, *t* test). (**O**) Representative LNCaP xenograft tumors from control and EtOH-fed mice. (**P**) The tumor growth is presented as a ratio of tumor weight (g)/number of days after injection; medians (min – max); N = 9 mice from each group; ***P < 0.05, unpaired t-test). (**Q**) ATF6 immunostaining (red) of the tissue sections from xenograft tumors presented in O. (**R**) Quantification of the ratio of nuclear/cytoplasmic ATF6 IF in cells presented in Q (means ± SD; N = 3; **P < 0.001, *t* test). All images were acquired with the same imaging parameters

The link between alcohol consumption and progression of PCa is well documented, but the mechanism is not fully understood [30–32]. Additionally, alcohol treatment is one of the best physiological models of ER stress. Chronic ethanol (EtOH) administration induces ER stress due to impaired maturation of proteins and their stalled export to the Golgi [33]. However, precisely how alcohol facilitates UPR, an important pathway of cancer survival [5, 34], is unknown. Our lab previously found that alcohol-induced Golgi disorganization was associated with the translocation of glycogen synthase kinase 3β (GSK3β) from the Golgi to the cytoplasm. This is significant to AR signaling in prostate cells because cytoplasmic GSK3β phosphorylates histone deacetylase 6 (HDAC6), which, in turn, activates heat shock protein 90 (HSP90) via its deacetylation. The deacetylated HSP90 is actively engaged in AR maturation and its transactivation [35]. Given the close relationship between ATF6 and AR described above, we proceeded to test the impact of alcohol on the S1P/S2P → ATF6 axis. In LNCaP cells, EtOH-induced Golgi fragmentation was associated with significant cleavage of ATF6 and its translocation into the nucleus (Fig. 6A-C, *P* < 0.001, *t* test). In EtOH-treated cells, we detected a decline in the expression of GCC185 and the development of ER stress, which was indicated by the elevated expression of GRP78 (Fig. 6D) and ATF6-dependent proteins HSP90 and calreticulin (Fig. 6E). In addition, a significantly larger amount of S1P and S2P was detected in the ER fraction of these cells (Fig. 6F and G). This was further validated by IF analysis: colocalization of both S1P and S2P with GCC185 was strongly reduced after EtOH administration (Fig. 6H-J). We also found that depletion of ATF6 in LNCaP cells blocks EtOH-induced intranuclear translocation of AR (Fig. 6K), which was associated with a reduction of both HDAC6 phosphorylation and deacetylation of HSP90 (Fig. 6L). The same phenomenon was also found in 22Rv1 cells (Fig. S7).

These data indicate the contribution of ATF6-mediated ER stress in EtOH-induced disorganization and activation of the GSK3β → HDAC6-P → HSP90 → AR axis. Then, we estimated the proliferation rate by anchorage-independent analysis. As shown in Fig. 6M and N, EtOH-treated cells transfected with control siRNAs produced a significantly higher number of colonies than cells treated with only control siRNAs; however, the growth of EtOH-treated ATF6-depleted cells was identical to the control cells. Further, we found that EtOH-treated LNCaP cells demonstrate enhanced migration through a Transwell chamber. However, such an effect of EtOH was abolished in cells lacking ATF6 (Fig. S8A-C). Previously, we have reported that LNCaP derived xenograft tumors are more prominent in alcohol-fed mice compared to their control counterparts [21]. Here, we reproduced these data (Fig. 6O and P, *P* < 0.01, *t* test) to evaluate the level of intranuclear ATF6. We found that in the nuclei of xenograft tumor cells from alcohol-fed mice, the intensity of the ATF6’s IF was elevated compared to the control group (Fig. 6Q and R, *P* < 0.001, *t* test).

**Fig. 7:**
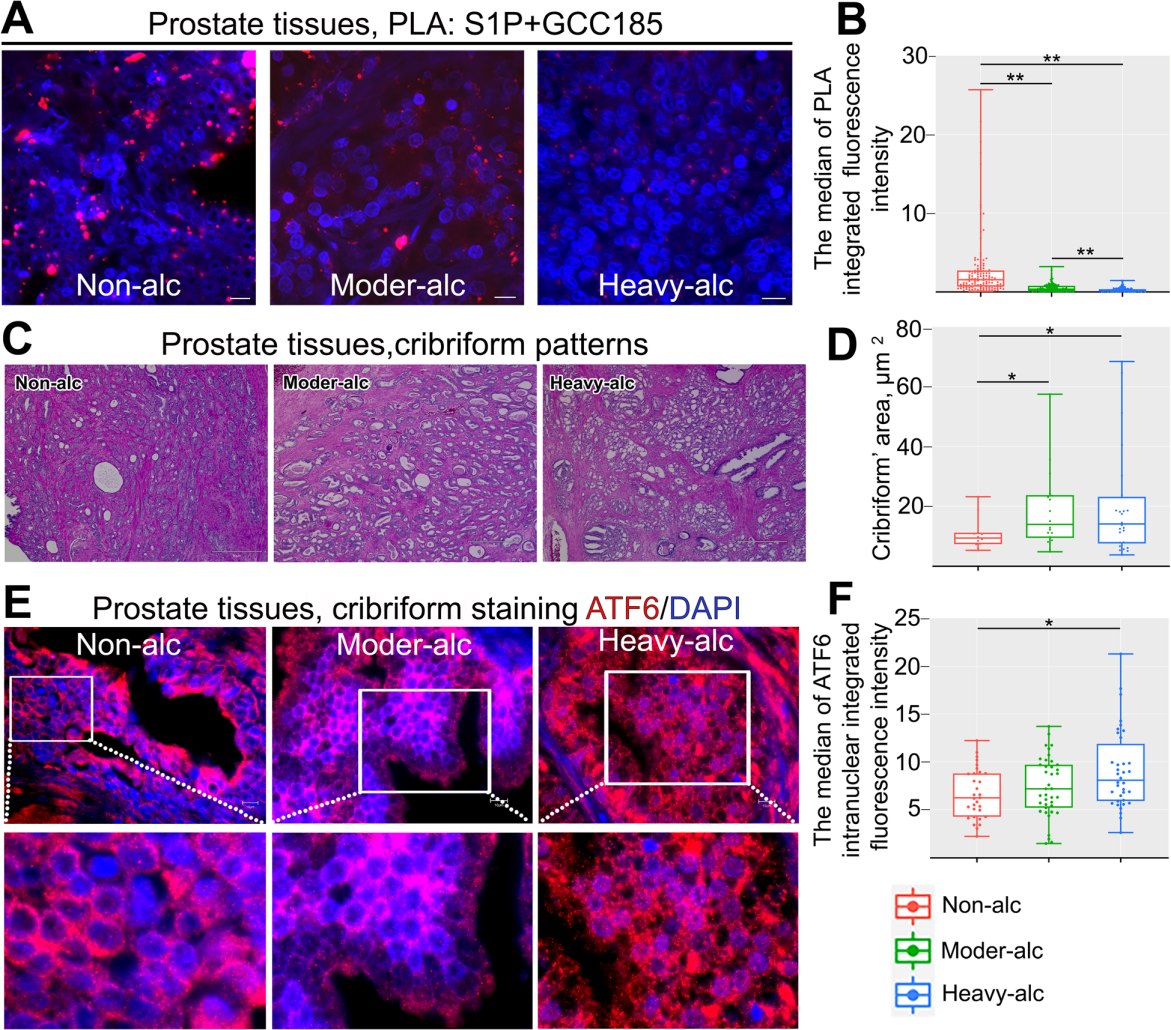
ATF6-mediated ER stress in patients with different rates of alcohol consumption. (**A**) Tissue sections from PCa patients with a Gleason score 7 were subjected to PLA using a combination of S1P/GCC185 Abs. Representative images from these patients are presented; all images were acquired with the same imaging parameters; bars, 10 μm. (**B**) Quantification of the PLA signal from samples presented in A. (**P < 0.01, pairwise Wilcoxon with Bonferonni-Hochberg multiple test; *N* = 14 for each group of patients). (**C**) The hematoxylin and eosin stain of the cribriform patterns from PCa patients with different alcohol consumption levels but the same Gleason score 7 (4 + 3 or 3 + 4); bars, 50 μm. (**D**) Quantification of the area of cribriform from samples presented in C (*P < 0.05, Mann-Whitney test). (**E**) Immunostaining of ATF6 (red) in the representative tissues from samples shown in C. White boxes indicate area magnified below; bars, 10 μm. (**F**) Quantification of ATF6 intranuclear intensity in cells from E (*P < 0.05, Mann-Whitney test). In B, D, and F, data are presented as medians (min – max)

Previously, we found that tumor tissue from PCa patients with the same stage of disease displayed different levels of Golgi fragmentation, which was positively correlated with the rate of alcohol consumption (see the Methods section for the definition of moderate and heavy levels of alcohol consumption) [21, 36]. This suggested that the segregation of S1P from GCC185, which was significantly different in tumors from patients with distinct Gleason scores (Fig. S4B and D), might be more visible in alcoholic patients compared to patients with the same stage of the disease but without alcohol addiction in their history. Indeed, screening of the PLA signal between S1P and GCC185 indicated that patients with moderate and high alcohol intake demonstrated a low degree of proximity of these proteins compared to non-alcoholic patients (Fig. 7A and B, *P* < 0.01, pairwise Wilcoxon with Bonferonni-Hochberg multiple test). The difference in the PLA signal between these two groups of alcohol-consuming patients was also significant (Fig. 7A and B, *P* < 0.01, pairwise Wilcoxon with Bonferonni-Hochberg multiple test). Also, it is known that PCa patients with cribriform growth at Gleason grade 4 have a worse prognosis than those with ‘poorly formed glands’ [37]. Here, we found that the size of the cribriform in patients with Gleason score 7 (4 + 3 or 3 + 4), which consumed alcohol at the high and moderate levels, was larger than that in non-alcoholic patients (Fig. 7C and D, *P* < 0.05, Mann-Whitney test). Next, we tested whether a difference between these cribriform patterns can also be detected in the ATF6 signaling. We measured the level of intranuclear ATF6 IF signal in cribriform patterns and found a significant difference between the groups of non-alcoholic and heavy drinkers; however, non-alcoholic patients did not differ from the patients who moderately consumed alcohol (Fig. 7E and F, *P* < 0.05, Mann-Whitney test).

It is known that ER stress has a profound effect on the central metabolic processes [38]. Here, we analyzed whether alcohol-induced ER stress is associated with significant perturbations of cellular pathways and whether these metabolic fluctuations are linked to the ATF6-mediated pathway. Using two-dimensional (2D) nuclear magnetic resonance (NMR) metabolomic profiling (2D ^1^H-^13^C HSQC), we detected a total of 106 spectral features across all cell media samples (five repeats for control and EtOH-treated LNCaP cells). Iterative class PCA models were calculated to identify potential outliers, with all control and EtOH-treated samples projected within the respective 95% confidence interval (CI). Supervised OPLS-DA analysis could differentiate control samples from EtOH-treated through one predictive component and one orthogonal component with R^2^_Y_ = 0.846, Q^2^_Y_ = 0.562 (7-fold cross-validation) and *P* = 0.026 (1000 permutations) (Fig. S9). The variables responsible for the separation could be attained using VIP scores, values greater than one (average) with significance, and were associated with 36 NMR features.

Searches across available databases/repositories – Human Metabolome Database (HMDB), Chenomx, Platform for RIKEN Metabolomics (PRIMe) – identified discriminatory NMR features associated with 20 unique metabolites. The intake of phenylalanine, N-Acetyl-beta-D-glucosamine, 2-oxoglutarate/α-ketoglutarate, glutamine, glutamate, acetate, N-acetylglutamine, lysine, and valine were observably increased in EtOH-treated samples. Conversely, the consumption of serine, glyceraldehyde/glycerose, cystine, tyrosine, ornithine, asparagine, 2-oxobutyrate/α-ketobutyrate, citrate, alanine, ethyl-oxaloacetate, and isoleucine were observably increased in control samples (Table S1).

In ATF6 KD cells, the consumption of glycine was reduced (Table S2). This would be expected given the lower proliferation rate of these cells (Fig. 4D-G), and the critical role of glycine in cancer cell growth, and the progression of PCa [39, 40]. Conversely, depletion of ATF6 in EtOH-treated cells reduced the consumption of pantothenic acid and the secretion of isocitric and lactic acids compared to cells administered with only EtOH. Additionally, the level of S-adenosyl-methionine, a universal methyl donor for protein and DNA methyltransferase reactions, was reduced in ATF6 KD cells treated with EtOH. Intriguingly, EtOH-induced production of aminoadipic acid, one of the markers of PCa recurrence [41] and γ-aminobutyric acid (GABA), which is upregulated with the onset of CRPC [42], was depressed in cells lacking ATF6 (Table S2).

A list of predicted intermediates – modules, enzymes, and reactions – from FELLA PT analysis were mapped to known genes (64 proteins / 102 genes) and used to perform Gene Ontology (GO) analysis (Table S3) of EtOH-treated cells. GO analysis for biological processes (BP), molecular functions (MF), and cellular components (CC) used clusterProfiler with Ensemble (org. Hs.eg.db), the hypergeometric test, and BH correction. GO analysis for BP exhibited enriched proteolysis, alpha, and cellular amino acid metabolism, catabolism, and biosynthesis, homeostasis (ion, cation, inorganic ion, cellular metal ion, cellular cation, and cellular ion), ATP hydrolysis- coupled cation transmembrane transport, multicellular organismal signaling, blood circulation, and circulatory system process (q-value < 0.001, threshold = 20). GO analysis for MF exhibited enriched peptidase activity (exo−/metallo−/metalloexo−/serine-type), transferase activity, transaminase activity, ATPase activity (transmembrane ion & substance moving, substance moving and cation transporting), ion activity, and cation transmembrane transporter activity (q-value < 0.001, threshold = 20). GO analysis for CC exhibited an enriched cytoplasmic vesicle, mitochondrial matrix (both are integral and intrinsic components of the plasma membrane), transporter, and transmembrane transporter complex (q-value < 0.05, threshold = 10).

## Discussion

**Fig. 8:**
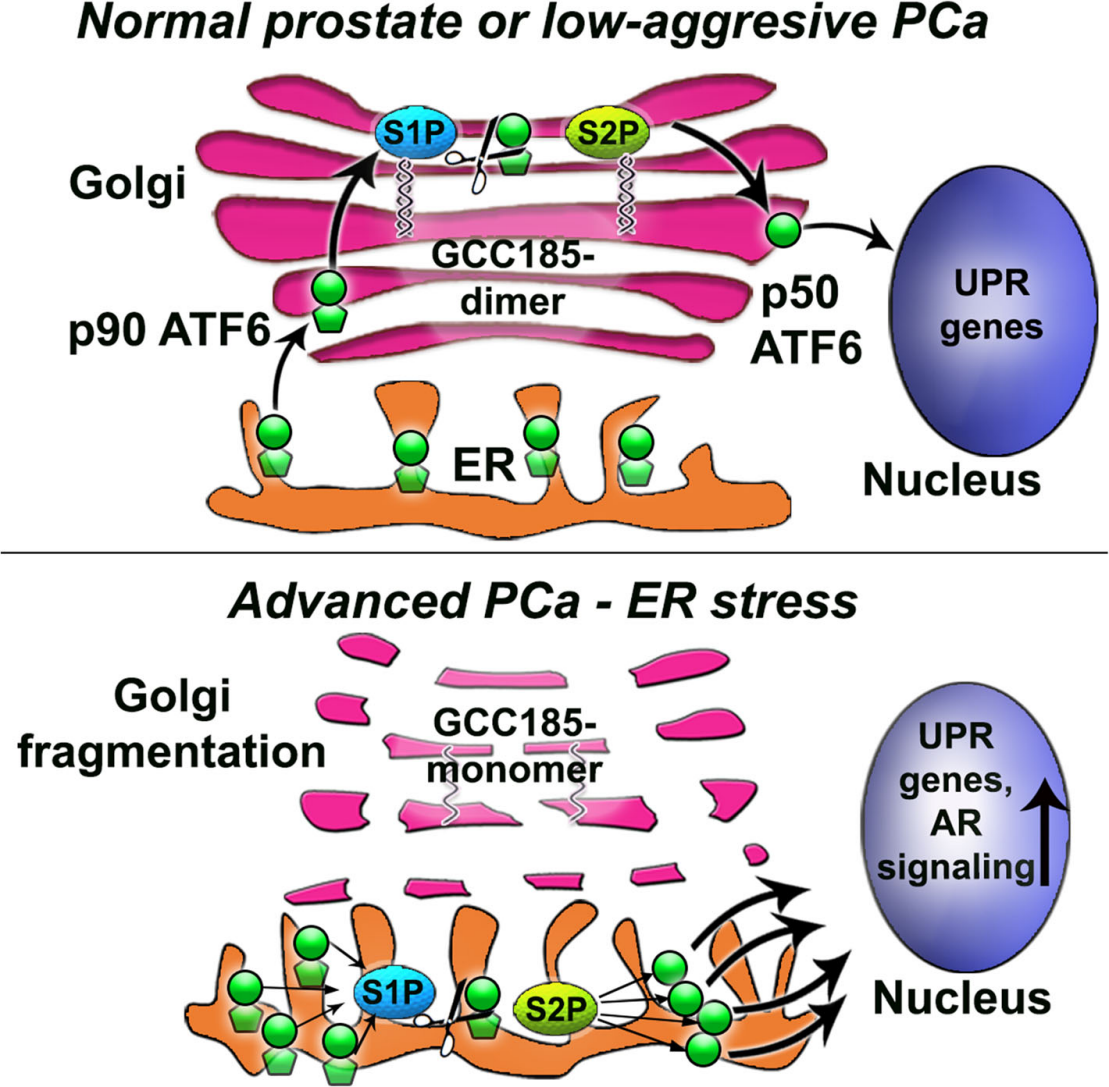
The working model of the self-activating mechanism of ER stress-mediated survival in PCa cells. (**A**) ATF6α is a 90 kDa type II transmembrane glycoprotein, which is transferred to the Golgi in normal prostate and low-aggressive PCa cells, where it undergoes cleavage by two proteases, S1P and S2P. These proteases are retained in the *trans*-Golgi by the dimeric form of GCC185. S1P and S2P sequentially remove the luminal domain and the transmembrane anchor of ATF6, respectively, mobilizing a 50 kDa N-terminal cytoplasmic fragment p50, which, in turn, enters the nucleus and binds to ER stress-response elements, stimulating the expression of UPR mediators. Thus, the number of ATF6 molecules transported to the Golgi correlates with the level of UPR. (**B**) In advanced PCa cells, Golgi’s disorganization is associated with the monomerization of GCC185, evoking translocation of both S1P and S2P to the ER and subsequent excessive cleavage of ATF6 molecules. Subsequently, high UPR signaling provides sufficient expression of chaperones that are required for AR-mediated tumor cell growth and proliferation

Our study uncovered a previously unexplored mechanism of how prostate cancer cells sustain ER stress injury in the face of Golgi disorganization. We found that S1P and S2P proteases relocate to the ER, thus obviating the need for ATF6 transport to the Golgi. This facilitates the cleavage of ATF6 and subsequent UPR, thereby accelerating cell proliferation (Fig. 8). Indeed, stable depletion of ATF6 significantly reduces tumor xenograft growth in vivo. In addition, we found that the segregation of both S1P and S2P from Golgi is correlated with an increase in Gleason score. Notably, in LNCaP cells, ER stress induced by Tg mimics the morphological features of PC-3 cells by induction of Golgi fragmentation and the shift of S1P and S2P from Golgi to the ER. These data explicitly prove the reciprocal link between ER stress and Golgi disorganization on one side [43] and ER stress and survival/proliferation signaling on the other [44]. It is important to note here that we detected an increase of both S1P and S2P expression in PCa tumors, which was significantly higher than that in BPH or NAT. This is rational as an accelerated cleavage of a substrate (ATF6) requires a corresponding increase in enzymes (S1P and S2P).

Our findings corroborate other researchers’ publications, suggesting that S1P and S2P may act in the ER after ER and Golgi stresses induced by Brefeldin A [22, 23], albeit the ER retention mechanism for these enzymes remains to be explored. Here, we identified *trans*-Golgi dimeric protein GCC185 as the Golgi retention partner for S1P and S2P. While we cannot exclude the contribution of other players, for instance, Rab and ARF proteins, to the situation of these proteases in the Golgi, our data unequivocally pointed to the direct interaction of both S1P and S2P with GCC185. Contrary to S1P and S2P, the expression of this golgin was reduced in PC-3 cells and the PCa tissue with a high Gleason score, implying the potential use of S1P, S2P, and GCC185 as the prognostic markers for this disease. Moreover, AFM analysis showed that in PC-3 cells, GCC185 persists in the Golgi in its monomeric form. On the one hand, this is an indication of events associated with ER stress, when stalled ER-to-Golgi trafficking may block posttranslational modification of Golgi matrix proteins, including their dimerization [24, 25, 45]. On the other hand, depletion of GCC185 may, in fact, induce Golgi disorganization [46]. However, this vicious circle, Golgi fragmentation↔ER stress↔downregulation of GCC185, does not lead to cell death, implying that PCa cells can trigger a tolerable level of ER stress inter alia by minimizing the contribution of Golgi proteins, such as GCC185, to the critical intracellular events, including apoptosis [47]. It this case, the degree of ER stress would be sufficient for tumor growth but not for the initiation of cell death.

This study provides compelling evidence that alcohol consumption can contribute to the development of sublethal ER stress in PCa cells. The effect of EtOH-induced Golgi disorganization echoes the results observed in ER stressed cells: Golgi disorganization, downregulation of GCC185, increase of GRP78 and cleaved ATF6, and translocation of S1P and S2P to the ER. The size of the xenograft tumor in EtOH-fed mice is larger than in the control group, as well as the expression of intranuclear ATF6. Our metabolomics data indicate that EtOH specifically activates several pro-oncogenic pathways, which are blocked upon the depletion of ATF6. Thus, the present study sheds new light on the crosstalk between alcohol-induced ER stress and cancer cells’ ability to survive and grow.

Finally, for the first time, we observed that synthesis of AR and its transactivation significantly relies on the ATF6-mediated UPR: in PCa tissue samples, which exhibit fragmented Golgi phenotype, we found a strong positive correlation between the expression of ATF6 and intranuclear AR. By shifting S1P and S2P to ATF6 and simplifying the ER stress pathway, cancer cells can rapidly provide an appropriate level of UPR to compensate for the need for AR protein itself and subsequent AR-triggered proliferation. In light of our recent publication indicating a link between Golgi fragmentation and the transactivation of AR [21], this seems logical. However, we do not rule out that hormone-induced expression of AR may reciprocally activate cleavage of ATF6, because androgens are able to induce dissociation of GRP78 from ATF6 [48]. An important question that arises is whether the link between ATF6 and AR exists in PCa patients after androgen deprivation therapy, given that their tumor still abundantly express AR [28]. Understanding the molecular mechanisms behind ER stress and AR signaling, including the contribution of the other two branches of UPR, IRE1α and PERK, can aid in the development of new therapeutic strategies that may use chemicals to alleviate ER stress in addition to AR blockade.

## Conclusions

We found that advanced PCa cells utilize an alternative mechanism of ATF6-mediated UPR, thus simplifying stress response and promoting the maturation of proteins responsible for the growth of the prostate tumor, including those involved in AR signaling. Activation of ATF6 correlates with the severity of this pathology and level of alcohol consumption. Depletion of ATF6 significantly blocks the growth of a prostate tumor, suggesting its potential implication in PCa therapy.

## Supplementary Information

**Additional file 1: Fig. S1.** (**A, B**). IF staining of RWPE-1 cells to detect colocalization of (**A**) S1P (green) and (**B**) S2P (green) with different *trans*-Golgi markers: GCC185 (red), GCC88 (red), TMF (red), and Golgin-245 (red). All images were acquired with the same imaging parameters, nucleus – blue, DAPI; bars, 10 μm. White boxes indicate the cell enlarged and shown at the right. (**C**) Quantification of the Pearson coefficient of colocalization for the cells presented in A and B (*N* = 90 cells from three repeats; **P < 0.001, *P < 0.01, *t* test). (**D**) GCC185, GCC88, TMF, and Golgin-245 W-B of the protein complexes from S1P and S2P IP samples prepared from RWPE-1 cells. **Fig. S2.** (**A**) Morphological staining of the Golgi by giantin in control and Tg-treated RWPE-1 and LNCaP cells, and non-treated PC-3 cells; bars, 10 μM. (**B**) Quantification of percent of cells with disorganized Golgi from A (*N* = 90 cells from three repeats; **P < 0.001, *t* test). (**C**) GRP78 W-B of RWPE-1, LNCaP (non-treated and Tg-treated), and PC-3 cell lysates; β-actin as a loading control. (**D, E**) S1P and S2P W-B of the ER (**D**) and Golgi (**E**) fractions isolated from LNCaP cells: control and Tg-treated. HSP70 and GM130 were used as a loading control for the ER and Golgi, respectively. **Fig. S3.** (**A**) S1P and S2P antibody was validated in the lung cancer tissue samples according to manifucture’s (Abcam) recommendation. (**B, D**) Immunohistochemical staining of S1P (**B**) and S2P (**D**) on the tissue samples from BPH and PCa patients. At least five representative areas were selected from the tumor area and normal tissue adjacent to tumor (NAT). Red boxes indicate the area enlarged and shown below. (**C, E**) Quantification of the expression of S1P (**C**) and S2P (**E**), presented as a ratio of the total intensity to the area (mm^2^). The details are described in the Methods section. Data are presented as medians (min – max); **P < 0.001, *P < 0.01, Mann-Whitney test. The number of patients counted for S1P: NAT – 10, BPH – 8, and PCa – 6; for S2P: NAT – 8, BPH – 11, and PCa – 7. **Fig. S4.** (**A**) Processing of PLA images using ImageJ software. **Fig. S5**. (**A-F**) Immunostaining of ATF6 (green) and AR (red) in the tissue: (**A**) normal prostate (**B**) Normal prostate tissue Adjacent Tumor (NAT), (**C**) BPH, (**D**) BPH with Low Grade Prostatic Intraepithelial Neoplasia (BPH LG PIN), (**E**) BPH with High Grade Prostatic Intraepithelial Neoplasia (BPH HG PIN), and (**F**) PCa. All images were acquired with the same imaging parameters, nucleus – blue, DAPI; bars 10 μm. Using ImageJ, each nucleus was outlined using the DAPI channel. **Fig. S6**. (**A, B**) Immunostaining of ATF6 (green) and AR (red) in LNCaP (**A**) and 22Rv1 (**B**) cells. All images were acquired with the same imaging parameters, nucleus – blue, DAPI; bars, 10 μm. **Fig. S7**. (**A**) AR W-B of the nuclear fraction from 22Rv1 cells: control, treated with ETOH, and ATF6 siRNA followed by EtOH. (**B**) HDAC6-P and HSP90-Ac W-B of the lysate of cells from A. **Fig. S8**. (**A**) Migration of LNCaP cells: control, treated with 50 mM EtOH for 96 h, and treated with EtOH in the presence of 100 nM ATF6 siRNAs; bars, 200 μm. Cell migration was measured via the Transwell chamber assay with an 8-μm pore size; an equal number of LNCaP cells (5 × 10^4^) in the three groups were seeded into the upper chamber with 200 μl of serum-free medium. (**B**) Quantification of the migrated cells for the cells presented in A (*N* = 90 cells from three repeats; **P < 0.001, t test). (**C**) ATF6 W-B of the lysate from scramble or ATF6 siRNA-transfected LNCaP cells. **Fig. S9**. Supervised OPLS-DA analysis (UV-scaled) between Ctrl and EtOH-treated samples of LNCaP cell media based on 106 metabolic features from 2D ^1^H-^13^C HSQC NMR spectra and created with MVAPACK (http://bionmr.unl.edu/mvapack.php) (Worley and Powers, 2014). (**A**) Scores plot (*n* = 10). (**B**) Model statistics – R^2^_Y_ = 0.846 and Q^2^_Y_ = 0.562 (7-fold CV). (**C**) VIP plot – 36 features (VIP > 1.0). (**D**) Permutation results (*n* = 1000) – *P* value = 0.026)). **Table S1:** List of identified metabolites from 36 discriminatory features (VIP > 1.0) from supervised OPLS-DA analysis between Ctrl and EtOH-treated samples of the LNCaP cells media based on 106 metabolic features from 2D ^1^H-^13^C HSQC NMR spectra. **Table S2:** List of discriminatory metabolites (*p*-value < 0.05) across conditions (Ctrl, ATF6 KD, EtOH-treated, and ATF6 KD/EtOH-treated) of LNCaP cell media based on 878 metabolic features acquired by 2D ^1^H-^13^C HSQC NMR experiments. **Table S3:** List of predicted intermediates (64 proteins / 102 genes) from KEGG ORA/PT pathway analysis with ‘FELLA’ and discriminatory NMR metabolites between Ctrl and EtOH-treated samples of the LNCaP cells media.

## Data Availability

The datasets used and/or analyzed during the current study are available from the corresponding author on reasonable request.

## Abbreviations

PCa: prostate cancer
ER: endoplasmic reticulum
ATF6: Activating Transcription Factor 6
AR: androgen receptor
EtOH: ethanol
PLA: proximity ligation assay

## References

[1] Stewart TA, Yapa KT, Monteith GR (2015). Altered calcium signaling in cancer cells. Biochim Biophys Acta.

[2] Liberti MV, Locasale JW (2016). The Warburg effect: how does it benefit Cancer cells?. Trends Biochem Sci.

[3] Moenner M, Pluquet O, Bouchecareilh M, Chevet E (2007). Integrated endoplasmic reticulum stress responses in cancer. Cancer Res.

[4] Oakes SA (2020). Endoplasmic reticulum stress signaling in Cancer cells. Am J Pathol.

[5] Yadav RK, Chae SW, Kim HR, Chae HJ (2014). Endoplasmic reticulum stress and cancer. J Cancer Prev.

[6] Mahadevan NR, Rodvold J, Sepulveda H, Rossi S, Drew AF, Zanetti M (2011). Transmission of endoplasmic reticulum stress and pro-inflammation from tumor cells to myeloid cells. Proc Natl Acad Sci U S A.

[7] Hung JH, Su IJ, Lei HY, Wang HC, Lin WC, Chang WT, et al. Endoplasmic Reticulum Stress Stimulates the Expression of Cyclooxygenase-2 through Activation of NF-κB and pp38 Mitogen-activated Protein Kinase. J Biol Chem. 2004;279(45):46384–92. 10.1074/jbc.M403568200. 10.1074/jbc.M403568200 15319438

[8] Katanasaka Y, Ishii T, Asai T, Naitou H, Maeda N, Koizumi F, Miyagawa S, Ohashi N, Oku N (2010). Cancer antineovascular therapy with liposome drug delivery systems targeted to BiP/GRP78. Int J Cancer.

[9] Shen J, Chen X, Hendershot L, Prywes R (2002). ER stress regulation of ATF6 localization by dissociation of BiP/GRP78 binding and unmasking of Golgi localization signals. Dev Cell.

[10] Martinon F (2012). Targeting endoplasmic reticulum signaling pathways in cancer. Acta Oncol.

[11] Storm M, Sheng X, Arnoldussen YJ, Saatcioglu F (2016). Prostate cancer and the unfolded protein response. Oncotarget..

[12] Guan M, Su L, Yuan YC, Li H, Chow WA (2015). Nelfinavir and nelfinavir analogs block site-2 protease cleavage to inhibit castration-resistant prostate cancer. Sci Rep.

[13] Sreenath TL, Macalindong SS, Mikhalkevich N, Sharad S, Mohamed A, Young D, Borbiev T, Xavier C, Gupta R, Jamal M, Babcock K, Tan SH, Nevalainen MT, Dobi A, Petrovics G, Sesterhenn IA, Rosner IL, Bieberich CJ, Nelson P, Vasioukhin V, Srivastava S (2017). ETS related gene mediated androgen receptor aggregation and endoplasmic reticulum stress in prostate Cancer development. Sci Rep.

[14] Mahadevan NR, Rodvold J, Almanza G, Perez AF, Wheeler MC, Zanetti M (2011). ER stress drives Lipocalin 2 upregulation in prostate cancer cells in an NF-kappaB-dependent manner. BMC Cancer.

[15] Egea G, Franci C, Gambus G, Lesuffleur T, Zweibaum A, Real FX (1993). cis-Golgi resident proteins and O-glycans are abnormally compartmentalized in the RER of colon cancer cells. J Cell Sci.

[16] Kellokumpu S, Sormunen R, Kellokumpu I (2002). Abnormal glycosylation and altered Golgi structure in colorectal cancer: dependence on intra-Golgi pH. FEBS Lett.

[17] McKinnon CM, Mellor H (2017). The tumor suppressor RhoBTB1 controls Golgi integrity and breast cancer cell invasion through METTL7B. BMC Cancer.

[18] Tan X, Banerjee P, Guo HF, Ireland S, Pankova D, Ahn YH, Nikolaidis IM, Liu X, Zhao Y, Xue Y, Burns AR, Roybal J, Gibbons DL, Zal T, Creighton CJ, Ungar D, Wang Y, Kurie JM (2017). Epithelial-to-mesenchymal transition drives a pro-metastatic Golgi compaction process through scaffolding protein PAQR11. J Clin Invest.

[19] Petrosyan A, Holzapfel MS, Muirhead DE, Cheng PW (2014). Restoration of compact Golgi morphology in advanced prostate cancer enhances susceptibility to galectin-1-induced apoptosis by modifying mucin O-glycan synthesis. Mol Cancer Res.

[20] Petrosyan A. Onco-Golgi: Is Fragmentation a Gate to Cancer Progression? Biochem Mol Biol J. 2015;1(1). 10.21767/2471-8084.100006. 10.21767/2471-8084.100006 PMC4824322 27064441

[21] Manca S, Frisbie CP, LaGrange CA, Casey CA, Riethoven JM, Petrosyan A (2019). The role of alcohol-induced Golgi fragmentation for androgen receptor signaling in prostate Cancer. Mol Cancer Res.

[22] Shen J, Prywes R (2004). Dependence of site-2 protease cleavage of ATF6 on prior site-1 protease digestion is determined by the size of the luminal domain of ATF6. J Biol Chem.

[23] DeBose-Boyd RA, Brown MS, Li WP, Nohturfft A, Goldstein JL, Espenshade PJ (1999). Transport-dependent proteolysis of SREBP: relocation of site-1 protease from Golgi to ER obviates the need for SREBP transport to Golgi. Cell..

[24] Frisbie CP, Lushnikov AY, Krasnoslobodtsev AV, Riethoven JJM, Clarke JL, Stepchenkova EI, et al. Post-ER Stress Biogenesis of Golgi Is Governed by Giantin. Cells. 2019;8:1631. 10.3390/cells8121631. 10.3390/cells8121631 PMC6953117 31847122

[25] Kim J, Noh SH, Piao H, Kim DH, Kim K, Cha JS, Chung WY, Cho HS, Kim JY, Lee MG (2016). Monomerization and ER Relocalization of GRASP is a requisite for unconventional secretion of CFTR. Traffic..

[26] Petrosyan A, Ali MF, Cheng PW (2012). Glycosyltransferase-specific Golgi-targeting mechanisms. J Biol Chem.

[27] Lonergan PE, Tindall DJ (2011). Androgen receptor signaling in prostate cancer development and progression. J Carcinog.

[28] Sadi MV, Walsh PC, Barrack ER (1991). Immunohistochemical study of androgen receptors in metastatic prostate cancer. Comparison of receptor content and response to hormonal therapy. Cancer..

[29] Cleutjens KB, van der Korput HA, van Eekelen CC, van Rooij HC, Faber PW, Trapman J (1997). An androgen response element in a far upstream enhancer region is essential for high, androgen-regulated activity of the prostate-specific antigen promoter. Mol Endocrinol.

[30] Zhao J, Stockwell T, Roemer A, Chikritzhs T (2016). Is alcohol consumption a risk factor for prostate cancer? A systematic review and meta-analysis. BMC Cancer.

[31] Middleton Fillmore K, Chikritzhs T, Stockwell T, Bostrom A, Pascal R (2009). Alcohol use and prostate cancer: a meta-analysis. Mol Nutr Food Res.

[32] Brunner C, Davies NM, Martin RM, Eeles R, Easton D, Kote-Jarai Z, al Olama AA, Benlloch S, Muir K, Giles G, Wiklund F, Gronberg H, Haiman CA, Schleutker J, Nordestgaard BG, Travis RC, Neal D, Donovan J, Hamdy FC, Pashayan N, Khaw KT, Stanford JL, Blot WJ, Thibodeau S, Maier C, Kibel AS, Cybulski C, Cannon-Albright L, Brenner H, Park J, Kaneva R, Batra J, Teixeira MR, Pandha H, Zuccolo L, the PRACTICAL Consortium (2017). Alcohol consumption and prostate cancer incidence and progression: a Mendelian randomisation study. Int J Cancer.

[33] Ji C (2012). Mechanisms of alcohol-induced endoplasmic reticulum stress and organ injuries. Biochem Res Int.

[34] Galmiche A, Sauzay C, Chevet E, Pluquet O (2017). Role of the unfolded protein response in tumor cell characteristics and cancer outcome. Curr Opin Oncol.

[35] Ai J, Wang Y, Dar JA, Liu J, Liu L, Nelson JB, Wang Z (2009). HDAC6 regulates androgen receptor hypersensitivity and nuclear localization via modulating Hsp90 acetylation in castration-resistant prostate cancer. Mol Endocrinol.

[36] Kubyshkin AV, Fomochkina II, Petrosyan AM (2018). The impact of alcohol on pro-metastatic N-glycosylation in prostate Cancer. Krim Z Eksp Klin Med.

[37] Iczkowski KA, Torkko KC, Kotnis GR, Wilson RS, Huang W, Wheeler TM (2011). Digital quantification of five high-grade prostate cancer patterns, including the cribriform pattern, and their association with adverse outcome. Am J Clin Pathol.

[38] Wang X, Eno CO, Altman BJ, Zhu Y, Zhao G, Olberding KE, Rathmell JC, Li C (2011). ER stress modulates cellular metabolism. Biochem J.

[39] Jain M, Nilsson R, Sharma S, Madhusudhan N, Kitami T, Souza AL, Kafri R, Kirschner MW, Clish CB, Mootha VK (2012). Metabolite profiling identifies a key role for glycine in rapid cancer cell proliferation. Science..

[40] Song YH, Shiota M, Kuroiwa K, Naito S, Oda Y (2011). The important role of glycine N-methyltransferase in the carcinogenesis and progression of prostate cancer. Mod Pathol.

[41] Jung K, Reszka R, Kamlage B, Bethan B, Stephan C, Lein M, Kristiansen G (2013). Tissue metabolite profiling identifies differentiating and prognostic biomarkers for prostate carcinoma. Int J Cancer.

[42] Taylor RA, Watt MJ (2019). Unsuspected Protumorigenic signaling role for the Oncometabolite GABA in advanced prostate Cancer. Cancer Res.

[43] Petrosyan A (2019). Unlocking Golgi: why does morphology matter?. Biochemistry (Mosc).

[44] Terai H, Kitajima S, Potter DS, Matsui Y, Quiceno LG, Chen T, Kim TJ, Rusan M, Thai TC, Piccioni F, Donovan KA, Kwiatkowski N, Hinohara K, Wei G, Gray NS, Fischer ES, Wong KK, Shimamura T, Letai A, Hammerman PS, Barbie DA (2018). ER stress signaling promotes the survival of Cancer "Persister cells" tolerant to EGFR tyrosine kinase inhibitors. Cancer Res.

[45] Casey CA, Thomes P, Manca S, Petrosyan A. Giantin Is Required for Post-Alcohol Recovery of Golgi in Liver Cells. Biomolecules. 2018;8:150. 10.3390/biom8040150. 10.3390/biom8040150 PMC6316505 30453527

[46] Derby MC, Lieu ZZ, Brown D, Stow JL, Goud B, Gleeson PA (2007). The trans-Golgi network golgin, GCC185, is required for endosome-to-Golgi transport and maintenance of Golgi structure. Traffic..

[47] Machamer CE (2003). Golgi disassembly in apoptosis: cause or effect?. Trends Cell Biol.

[48] Yang YC, Fu HC, Hsiao BL, Sobue G, Adachi H, Huang FJ, Hsuuw YD, Wei KT, Chang C, Huang KE, Kang HY (2013). Androgen receptor inclusions acquire GRP78/BiP to ameliorate androgen-induced protein misfolding stress in embryonic stem cells. Cell Death Dis.

