# Supplementary material for: The non-canonical mechanism of ER stress-mediated progression of prostate cancer
Source: J Exp Clin Cancer Res. 2021 Sep 14;40:289. doi: 10.1186/s13046-021-02066-7 (PMC8439065; doi:10.1186/s13046-021-02066-7)
Supplement: Supplementary file 1 — Additional file 1: Fig. S1. (A, B). IF staining of RWPE-1 cells to detect colocalization of (A) S1P (green) and (B) S2P (green) with different trans-Golgi markers: GCC185 (red), GCC88 (red), TMF (red), and Golgin-245 (red). All images were acquired with the same imaging parameters, nucleus – blue, DAPI; bars, 10 μm. White boxes indicate the cell enlarged and shown at the right. (C) Quantification of the Pearson coefficient of colocalization for the cells presented in A and B (N = 90 cells from three repeats; **P < 0.001, *P < 0.01, t test). (D) GCC185, GCC88, TMF, and Golgin-245 W-B of the protein complexes from S1P and S2P IP samples prepared from RWPE-1 cells. Fig. S2. (A) Morphological staining of the Golgi by giantin in control and Tg-treated RWPE-1 and LNCaP cells, and non-treated PC-3 cells; bars, 10 μM. (B) Quantification of percent of cells with disorganized Golgi from A (N = 90 cells from three repeats; **P < 0.001, t test). (C) GRP78 W-B of RWPE-1, LNCaP (non-treated and Tg-treated), and PC-3 cell lysates; β-actin as a loading control. (D, E) S1P and S2P W-B of the ER (D) and Golgi (E) fractions isolated from LNCaP cells: control and Tg-treated. HSP70 and GM130 were used as a loading control for the ER and Golgi, respectively. Fig. S3. (A) S1P and S2P antibody was validated in the lung cancer tissue samples according to manifucture’s (Abcam) recommendation. (B, D) Immunohistochemical staining of S1P (B) and S2P (D) on the tissue samples from BPH and PCa patients. At least five representative areas were selected from the tumor area and normal tissue adjacent to tumor (NAT). Red boxes indicate the area enlarged and shown below. (C, E) Quantification of the expression of S1P (C) and S2P (E), presented as a ratio of the total intensity to the area (mm2). The details are described in the Methods section. Data are presented as medians (min – max); **P < 0.001, *P < 0.01, Mann-Whitney test. The number of patients counted for S1P: NAT – 10, BPH – 8, and PCa – 6; f [file 13046_2021_2066_MOESM1_ESM.docx]

Supplementary Materials for

**The non-canonical mechanism of ER stress-mediated progression of prostate cancer**

**Authors:** Artem N. Pachikov, Ryan R. Gough, Caroline E. Christy, Mary E. Morris, Carol A. Casey, Chad A. LaGrange, Ganapati Bhat, Anatoly V. Kubyshkin, Iryna I. Fomochkina, Evgeniya Y. Zyablitskaya, Tatiana P. Makalish, Elena P. Golubinskaya, Kateryna A. Davydenko, Sergey N. Eremenko, Jean-Jack M. Riethoven, Amith S. Maroli, Thomas S. Payne, Robert Powers, Alexander Y. Lushnikov, Amanda J Macke, and Armen Petrosyan*

Correspondence to: [apetrosyan@unmc.edu](mailto:apetrosyan@unmc.edu)

**This PDF file includes:**

Figs. S1 to S9

Tables S1 to S3

MATERIAL AND METHODS


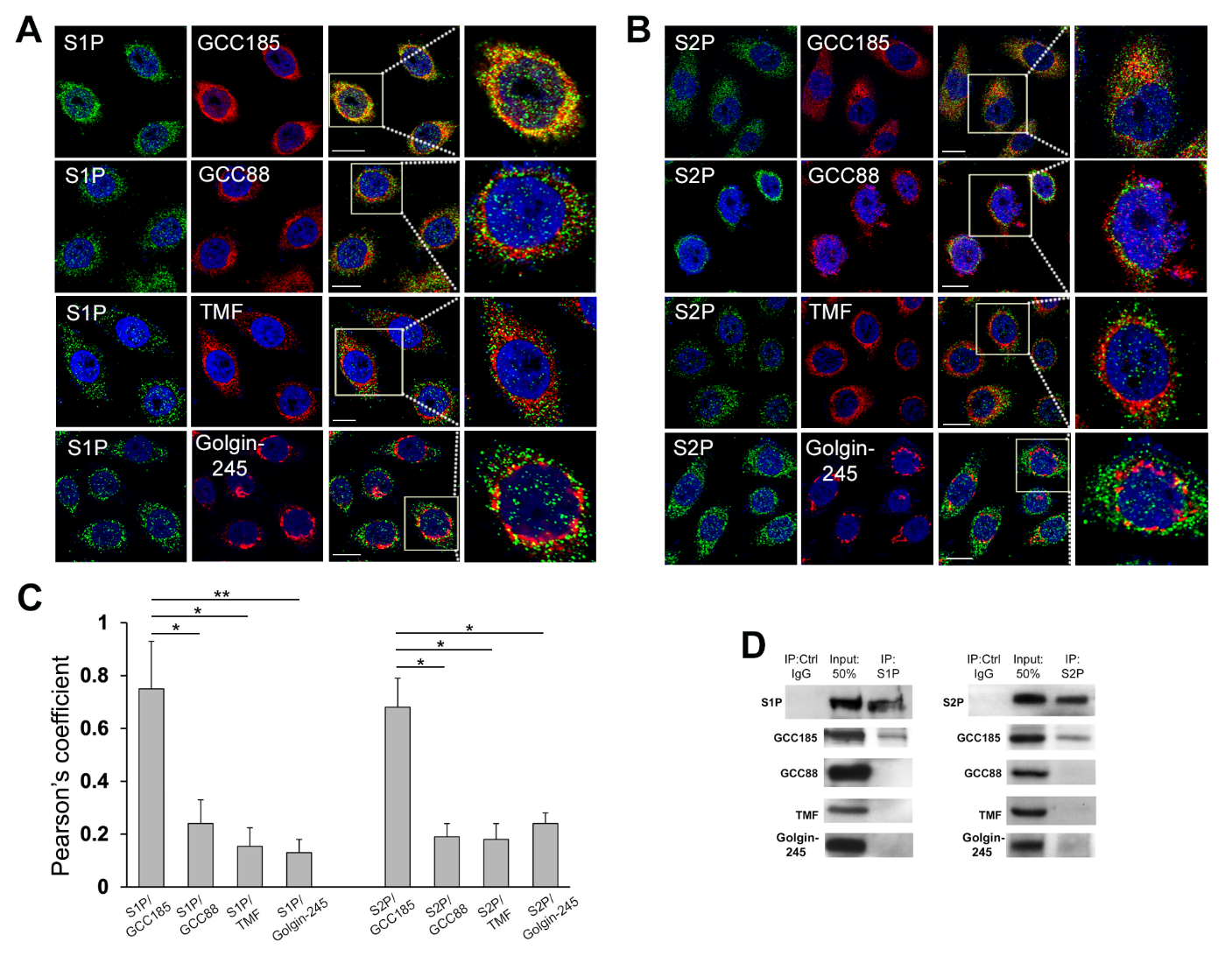


Figure S1. (A, B). IF staining of RWPE-1 cells to detect colocalization of (A) S1P (green) and (B) S2P (green) with different *trans*-Golgi markers: GCC185 (red), GCC88 (red), TMF (red), and Golgin-245 (red). All images were acquired with the same imaging parameters, nucleus – blue, DAPI; bars, 10 μm. White boxes indicate the cell enlarged and shown on the right. (C) Quantification of the Pearson coefficient of colocalization for the cells presented in A and B (*N* = 90 cells from three repeats; ***P* < 0.001, **P* < 0.01, *t* test). (D) GCC185, GCC88, TMF, and Golgin-245 W-B of the protein complexes from S1P and S2P IP samples prepared from RWPE-1 cells.


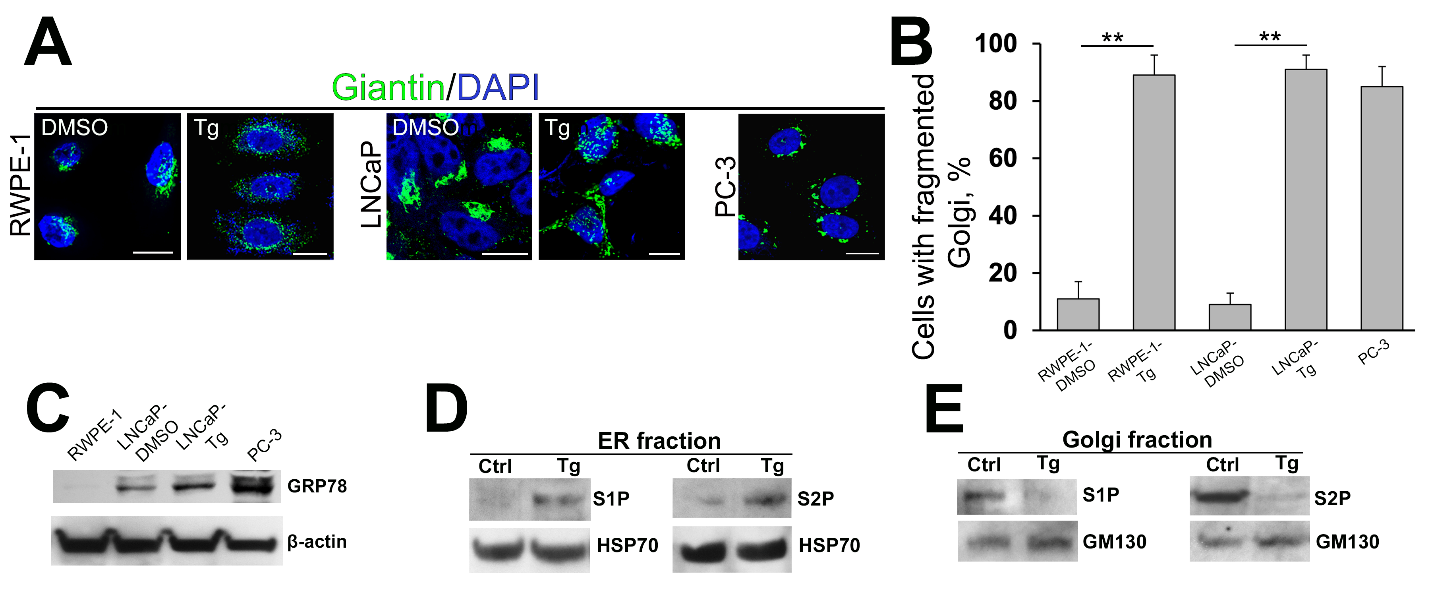
**Figure S2.** (A) Morphological staining of the Golgi by giantin in control and Tg-treated RWPE-1 and LNCaP cells, and non-treated PC-3 cells; bars, 10µM. (B) Quantification of percent of cells with disorganized Golgi from A (*N* = 90 cells from three repeats; ***P* < 0.001, *t* test). (C) GRP78 W-B of RWPE-1, LNCaP (non-treated and Tg-treated), and PC-3 cell lysates; β-actin as a loading control. (D, E) S1P and S2P W-B of the ER (D) and Golgi (E) fractions isolated from LNCaP cells: control and Tg-treated. HSP70 and GM130 were used as a loading control for the ER and Golgi, respectively.


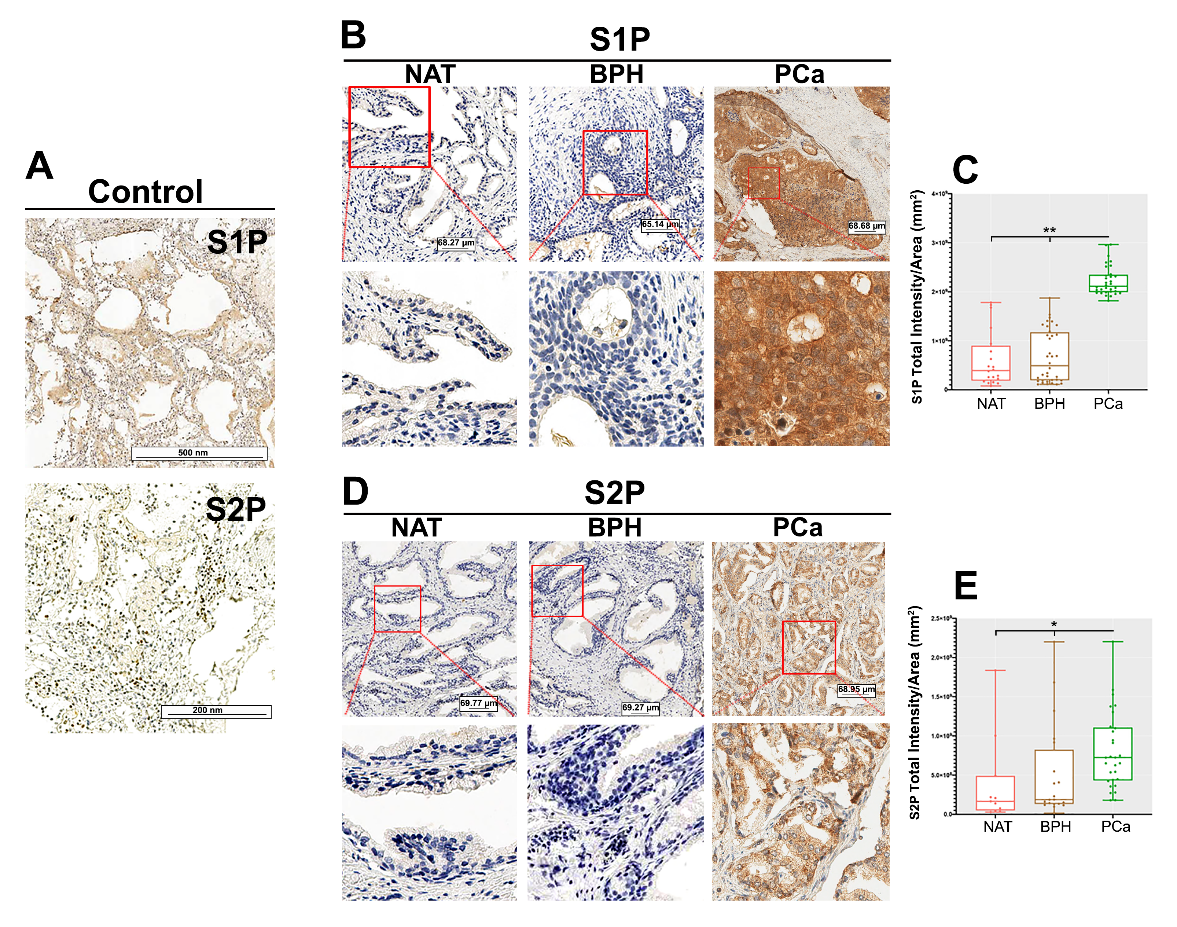


**Figure S3.** (A) S1P and S2P antibody was validated in the lung cancer tissue samples according to the manufacture’s (Abcam) recommendation. (B, D) Immunohistochemical staining of S1P (B) and S2P (D) on the tissue samples from BPH and PCa patients. At least five representative areas were selected from the tumor area and normal tissue adjacent to tumor (NAT). Red boxes indicate the area enlarged and shown below. (C, E) Quantification of the expression of S1P (C) and S2P (E), presented as a ratio of the total intensity to the area (mm^2^). The details are described in the Methods section. Data are presented as medians (min – max); ***P* < 0.001, **P* < 0.01, Mann-Whitney test. The number of patients counted for S1P: NAT – 10, BPH – 8, and PCa – 6; for S2P: NAT – 8, BPH – 11, and PCa – 7.


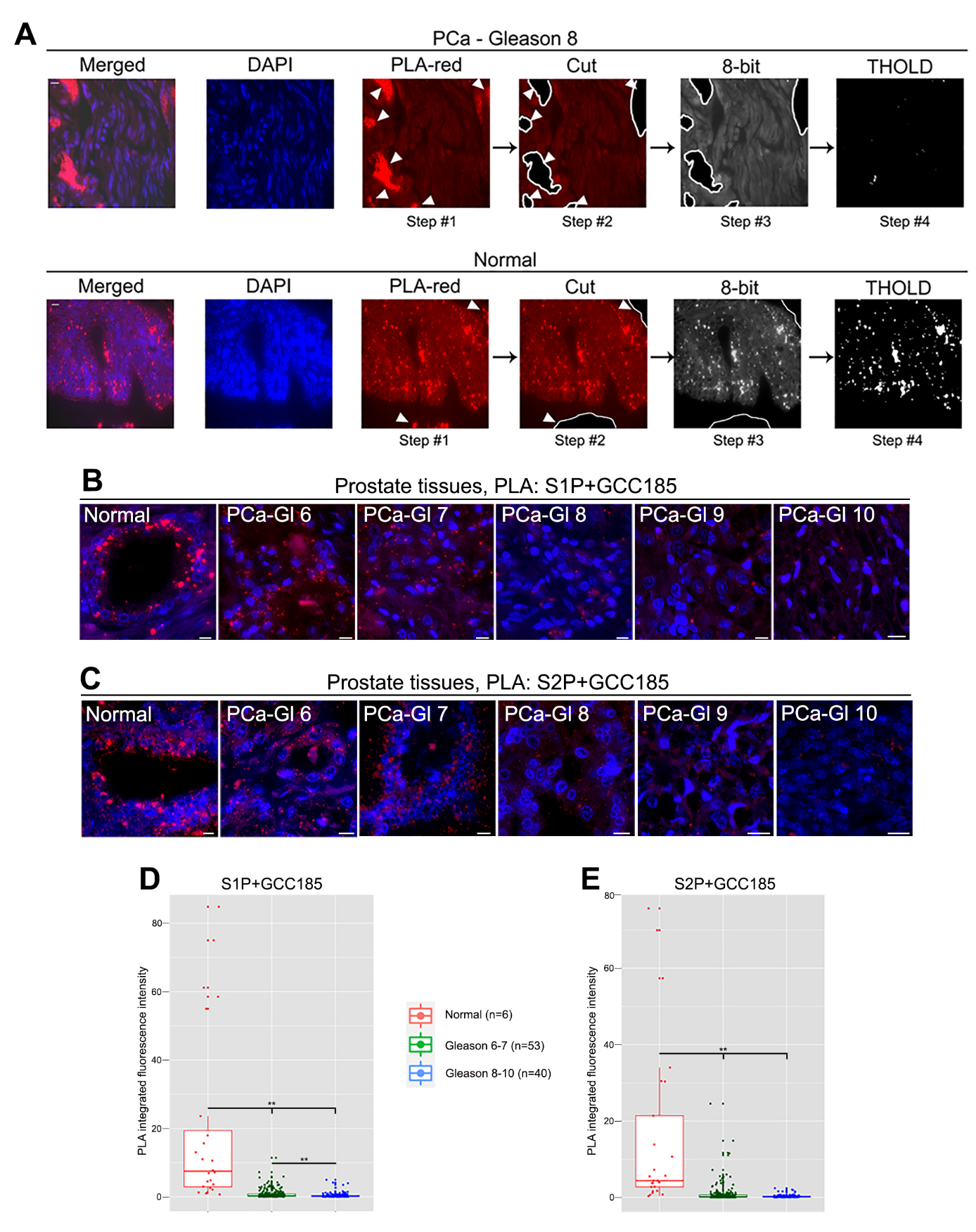


**Figure S4.** (A) Processing of PLA images using ImageJ software. Images were captured using the EVOS M5000 Imaging System (Thermo Fisher Scientific, USA) and processed by ImageJ. PLA signal is in the red channel and blue channel is DAPI – nucleus. Upper panel: The image with the lowest PLA signal was selected (PCa – Gleason 8), and areas of nonspecific binding (indicated by the white arrowheads in Step #1) were cropped (shown in Step #2). The image was then converted to 8-bit (Step #3) and the threshold (THOLD) was adjusted (Step #4) until the intensity of the PLA signal matched the area of PLA signal from the original red image (PLA-red). The threshold value was then applied to all images. Lower panel: The PLA-red channel image from normal prostate tissue was cut to remove nonspecific binding as in the upper panel (Steps #1&2), converted to 8-bit (Step #3), and a threshold was selected (THOLD) using the threshold determined in the upper panel (Step #4). All images were processed using the same method as described and acquired with the same imaging parameters; bars, 10 μm. (B, C) Confirmation of S1P and S2P segregation from the Golgi in PCa tissues using PLA. Tissue sections from normal prostate tissue and PCa patients with different Gleason scores were subjected to PLA utilizing a combination of (B) S1P/GCC185 and (C) S2P/GCC185 antibodies. Representative images from normal prostate and patients with Gleason scores from 6 to 10 are presented. All images were acquired with the same imaging parameters, nucleus – blue, DAPI; bars, 10 μm. (D, E): Quantification of the PLA signal from samples presented in B and C, respectively. For S1P/GCC185, a significant difference was observed between normal tissue and tumors when combining patients with Gleason scores 6-7 and 8-10 and between both groups of patients (***P* < 0.001, pairwise Wilcoxon with Bonferonni-Hochberg multiple test). For S2P/GCC185, a significant difference was also observed between normal tissue and tumors from patients when combining Gleason scores 6-7 and 8-10 (***P* < 0.001, pairwise Wilcoxon with Bonferonni-Hochberg multiple test); however, there was no significance found when comparing these two groups to each other.

**
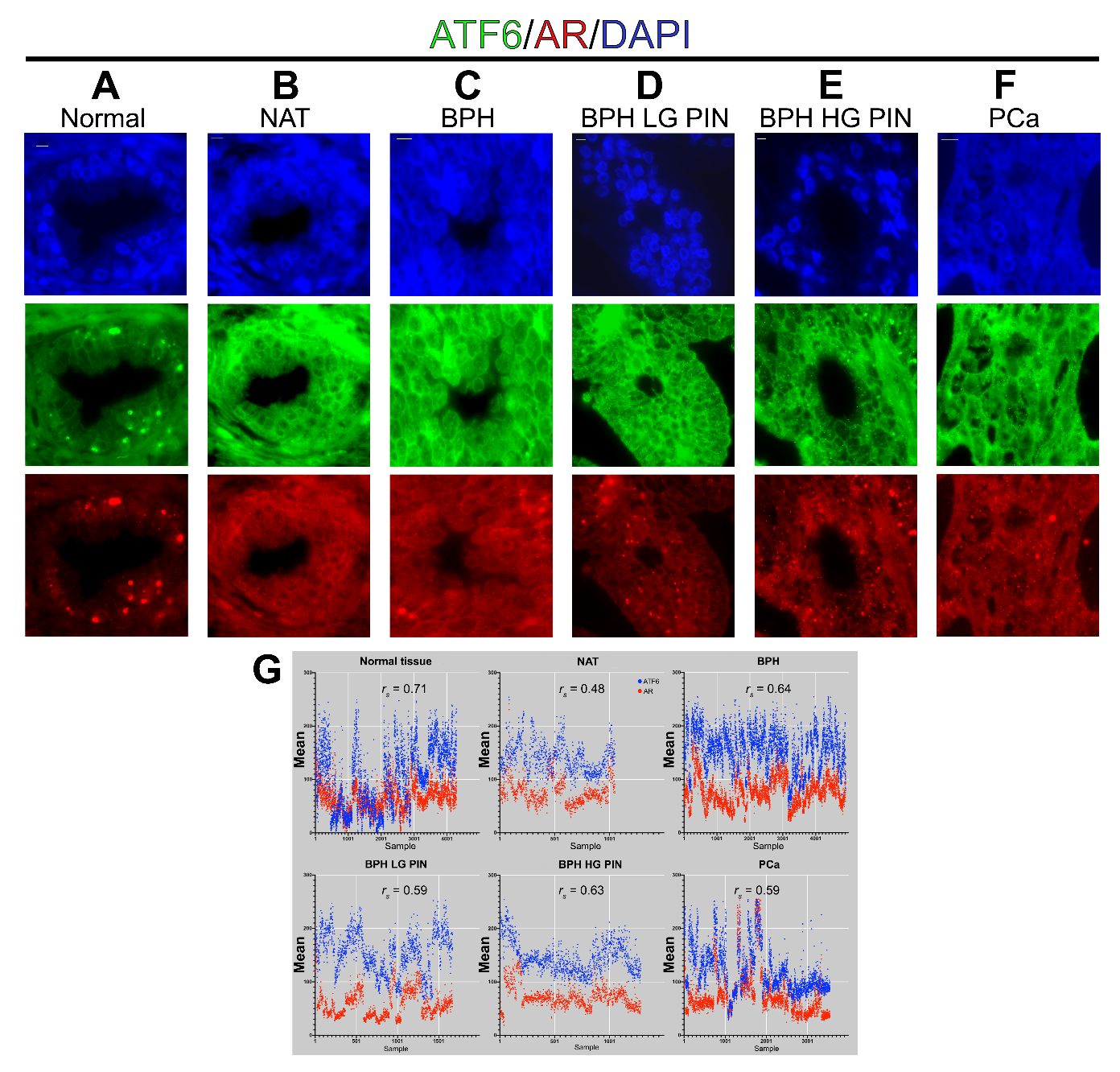
Figure S5**. (A-F) Immunostaining of ATF6 (green) and AR (red) in the tissue: (A) normal prostate (B) Normal prostate tissue Adjacent Tumor (NAT), (C) BPH, (D) BPH with Low Grade Prostatic Intraepithelial Neoplasia (BPH LG PIN), (E) BPH with High Grade Prostatic Intraepithelial Neoplasia (BPH HG PIN), and (F) PCa. All images were acquired with the same imaging parameters, nucleus – blue, DAPI; bars 10 µm. Using ImageJ, each nucleus was outlined using the DAPI channel. These outlines were saved as a Region of Interest (ROI) and applied to the corresponding green (ATF6) and red (AR) channels and used to measure the mean IF intensity. Prism version 8.0 (GraphPad) was used for further analysis of normality and correlation. (G) Correlation analysis between the intranuclear IF intensity of ATF6 (blue) and AR (red) in the tissues from A-F; (*P* < 0.001, Spearman Rank Correlation Coefficient, *r_s_*). The number of cells counted was ~4293 for normal tissue samples, ~1050 for NAT, ~4911 for BPH, ~1669 for BPH LG PIN, ~1286 for BPH HG PIN, and ~3546 for PCa.


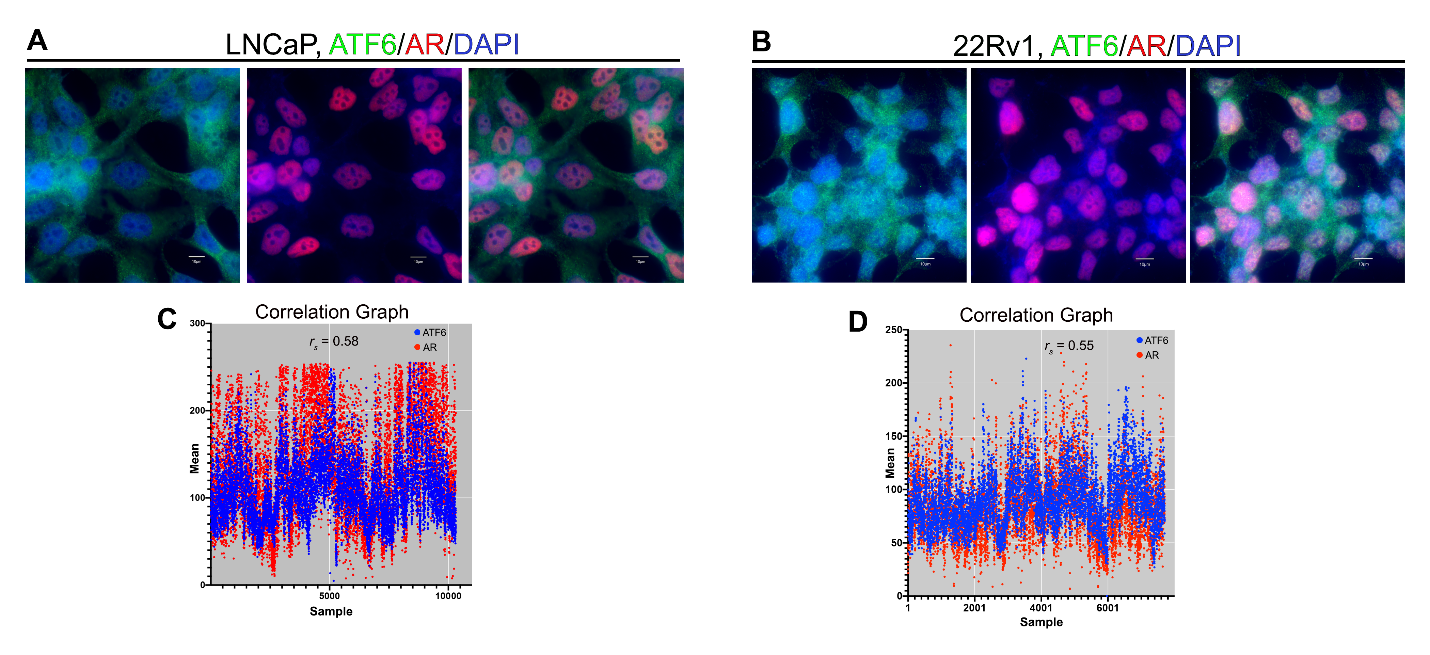


**Figure S6**. (A, B) Immunostaining of ATF6 (green) and AR (red) in LNCaP (A) and 22Rv1 (B) cells. All images were acquired with the same imaging parameters, nucleus – blue, DAPI; bars, 10 µm. Using ImageJ, each nucleus was outlined using the DAPI channel. These outlines were saved as a Region of Interest (ROI) and applied to the corresponding green (ATF6) and red (AR) channels and used to measure the mean IF intensity within the nuclei. Prism version 8.0 (GraphPad) was used for further analysis of normality and correlation. (C) Correlation analysis between intranuclear IF intensity of ATF6 (blue) and AR (red) (*P* < 0.001, Spearman Rank Correlation Coefficient, *r_s_*) for samples from A. Approximately 10,300 LNCaP cells were counted from three independent experiments. (D) Correlation analysis between the intranuclear IF intensity of ATF6 (blue) and AR (red) (*P* < 0.001, Spearman Rank Correlation Coefficient, *r_s_*) in samples from B. Approximately 7,700 22Rv1 cells were counted from three independent experiments.

**
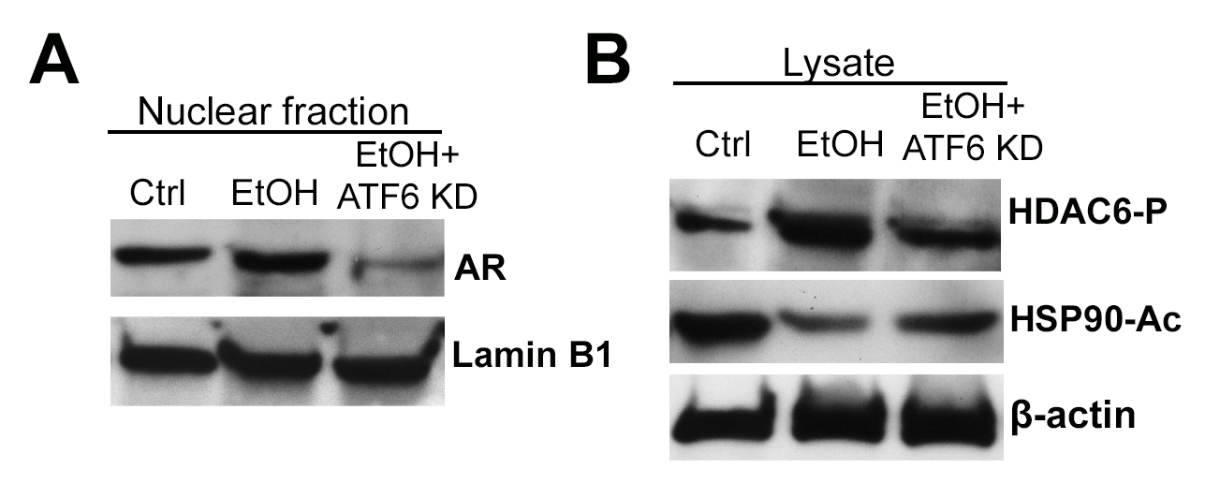
**

**Figure S7**. (A) AR W-B of the nuclear fraction from 22Rv1 cells: control, treated with ETOH, and ATF6 siRNA followed by EtOH. (B) HDAC6-P and HSP90-Ac W-B of the lysate of cells from A.


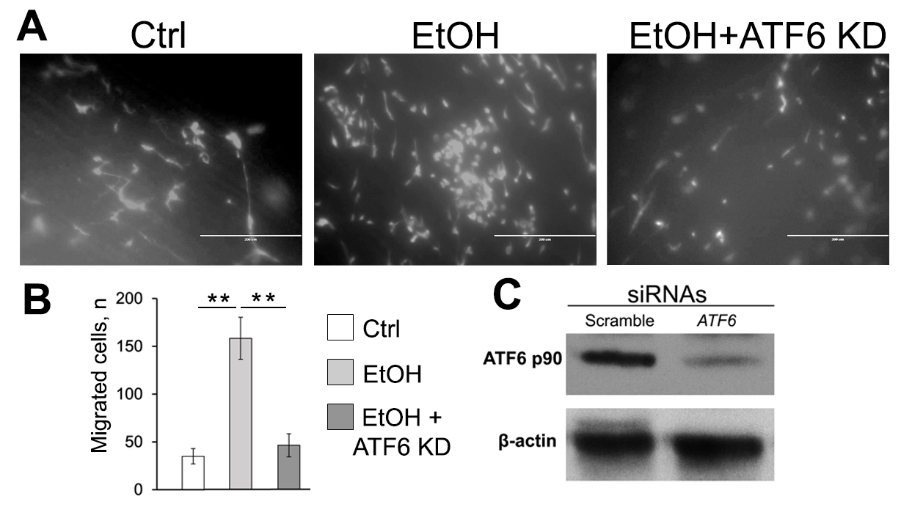


**Figure S8**. (A) Migration of LNCaP cells: control, treated with 50 mM EtOH for 96 h, and treated with EtOH in the presence of 100 nM ATF6 siRNAs; bars, 200 μm. Cell migration was measured via the Transwell chamber assay with an 8-μm pore size; an equal number of LNCaP cells (5×10^4^) in the three groups were seeded into the upper chamber with 200 µl of serum-free medium. (B) Quantification of the migrated cells for the cells presented in A (N = 90 cells from three repeats; ***P* < 0.001, t test). (C) ATF6 W-B of the lysate from scramble or ATF6 siRNA-transfected LNCaP cells.

**
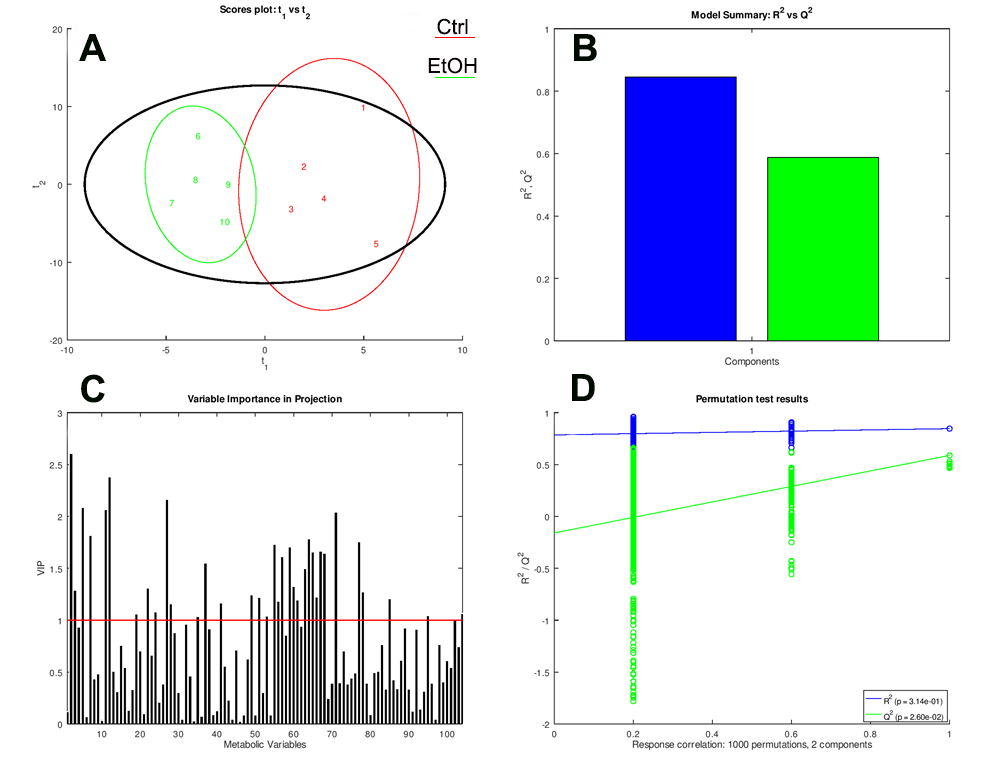
**

**Figure S9**. Supervised OPLS-DA analysis (UV-scaled) between Ctrl and EtOH-treated samples of LNCaP cell media based on 106 metabolic features from 2D ^1^H-^13^C HSQC NMR spectra and created with MVAPACK (http://bionmr.unl.edu/mvapack.php)(Worley and Powers, 2014). (A) Scores plot (n=10). (B) Model statistics – R^2^_Y_ = 0.846 and Q^2^_Y_ = 0.562 (7-fold CV). (C) VIP plot – 36 features (VIP > 1.0). (D) Permutation results (n=1000) – *P* value = 0.026)).

**Table S1:** List of identified metabolites from 36 discriminatory features (VIP > 1.0) from supervised OPLS-DA analysis between Ctrl and EtOH-treated samples of the LNCaP cells media based on 106 metabolic features from 2D ^1^H-^13^C HSQC NMR spectra.

|  | Name | KEGG-ID | HMDB-ID | ppm (1H, 13C) | Loadings (OPLS) | VIP (OPLS) |
| --- | --- | --- | --- | --- | --- | --- |
| 1 | L-Phenylalanine | C00079 | HMDB0000159 | 7.32, 132.11 \| 7.42, 131.95 | 0.1177 \| 0.0991 | 1.01 \| 1.07 |
| 2 | N-Acetyl-beta-D-glucosamine | C03878 | HMDB0000803 | 5.18, 92.58 | 0.0693 | 1.04 |
| 3 | L-Serine | C00065 | HMDB0000187 | 3.83, 59.52 | -0.0664 | 1.21 |
| 4 | Glyceraldehyde / Glycerose | C02154 | HMDB0001051 | 3.56, 65.89 \| 3.65, 65.93 | -0.1581 \|  -0.1300 | 1.75 \| 1.27 |
|  |  |  |  |  |  |  |
| 5 | L-Cystine | C00491 | HMDB0000192 | 3.15, 41.64 \| 3.35, 41.32 | -0.1951 \|  -0.2075 | 1.67 \| 2.04 |
| 6 | L-Tyrosine | C00082 | HMDB0000158 | 3.00, 38.11 \| 3.19, 38.00 | -0.1809 \|  -0.1770 | 1.65 \| 1.65 |
| 7 | L-Ornithine | C00077 | HMDB0000214 | 3.05, 41.91 | -0.129 | 1.22 |
|  |  |  |  |  |  |  |
| 8 | L-Asparagine | C00152 | HMDB0000168 | 2.84, 37.66 \| 2.93, 37.69 | -0.1756 \|  -0.2091 | 1.50 \| 1.78 |
| 9 | 2-Oxobutyrate / α-Ketobutyrate | C00109 | HMDB0000005 | 2.76, 35.53 | -0.0879 | 1.2 |
|  |  |  |  |  |  |  |
| X | 2-Oxohexanoate / α-Ketohexanoic acid | C00902 | HMDB0001864 | 2.72, 41.55 |  |  |
| X | Guanosine triphosphate (GTP) | C00044 | HMDB0001273 | 2.72, 41.55 |  |  |
|  |  |  |  |  |  |  |
| 10 | Citrate / Citric acid | C00158 | HMDB0000094 | 2.53, 47.87 \| 2.67, 48.20 | -0.0829 \|  -0.0971 | 1.04 \| 1.18 |
| 11 | 2-Oxoglutarate / α-Ketoglutarate | C00026 | HMDB0000208 | 2.44, 33.72 | 0.1153 | 1.24 |
|  |  |  |  |  |  |  |
| 12 | L-Glutamine | C00064 | HMDB0000641 | 2.12, 29.50 | 0.1671 | 1.55 |
| 13 | L-Glutamate | C00025 | HMDB0000148 | 2.05, 29.95 | 0.1441 | 1.04 |
| 14 | Acetate / Acetic Acid | C00033 | HMDB0000042 | 1.91, 26.09 | 0.1334 | 1.16 |
| 15 | N-Acetylglutamine |  | HMDB0006029 | 1.87, 31.50 | 0.2283 | 2.16 |
|  |  |  |  |  |  |  |
| 16 | L-Lysine | C00047 | HMDB0000182 | 1.71, 28.62 | 0.1049 | 1.31 |
| 17 | L-Alanine | C00041 | HMDB0000161 | 1.47, 18.99 | -0.0448 | 1.06 |
| 18 | Ethyl-oxaloacetate | NaN | NaN | 1.26, 15.56 | -0.2187 | 2.07 |
| 19 | L-Valine | C00183 | HMDB0000883 | 0.97, 20.27 \| 1.03, 20.57 | 0.1674 \| 0.1201 | 2.09 \| 1.82 |
| 20 | L-Isoleucine | C00407 | HMDB0000172 | 0.93, 13.92 | -0.1577 | 1.29 |

**Table S2:** List of discriminatory metabolites (p-value < 0.05) across conditions (Ctrl, ATF6 KD, EtOH-treated, and ATF6 KD/EtOH-treated) of LNCaP cell media based on 878 metabolic features acquired by 2D ^1^H-^13^C HSQC NMR experiments.

| **Name** | **KEGG-ID** | **HMDB-ID** | **Ppm (1H, 13C)** | **Significance** | **log2 FC**  **(LIMMA)** | **t.score (LIMMA)** | **p.value (LIMMA)** |
| --- | --- | --- | --- | --- | --- | --- | --- |
| glycine | C00037 | HMDB0000123 | 3.49, 84.16 | CTRL v ATF6KD | 7.09 | 3.03 | 0.0141 |
| L-2-aminoadipic acid | C00956 | HMDB0000510 | 2.28, 79.86 | CTRL v EtOH ATF6 KD/EtOH v EtOH | -8.71 -6.90 | -3.97 -2.27 | 0.0033 0.0490 |
| pantothenic acid | C00864 | HMDB0000210 | 2.18, 79.69 | ATF6KD/EtOH  v EtOH | -6.67 | -2.29 | 0.0208 |
| 4-aminobutyric acid | C00334 | HMDB0000112 | 1.92, 106.56 | ATF6KD/EtOH  v EtOH | -1.38 | -2.63 | 0.0272 |
| isocitric acid | C00311 | HMDB0000193 | 2.41, 40.41 | ATF6KD/EtOH  v EtOH | -8.34 | -4.24 | 0.0022 |
| D-lactic acid | C00256 | HMDB0001311 | 1.32, 22.53 | ATF6KD/EtOH  v EtOH | -1.74 | -2.28 | 0.0488 |

**Table S3:** List of predicted intermediates (64 proteins / 102 genes) from KEGG ORA/PT pathway analysis with ‘FELLA’ and discriminatory NMR metabolites between Ctrl and EtOH-treated samples of the LNCaP cells media.

|  | EC_number | p.score | EC_name | Gene ENTREZID | Gene ENSEMBL | Gene SYMBOL |
| --- | --- | --- | --- | --- | --- | --- |
| 1 | 3.4.17.23 | 0.00000 | angiotensin-converting enzyme 2 | 59272 | ENSG00000130234 | ACE2 |
| 2 | 6.3.5.4 | 0.00000 | asparagine synthase (glutamine-hydrolysing) | 440 | ENSG00000070669 | ASNS |
| 3 | 7.2.2.8 | 0.00000 | P-type Cu+ transporter | 538;540 | ENSG00000165240;ENSG00000123191 | ATP7A;ATP7B |
| 4 | 3.4.16.2 | 0.00000 | lysosomal Pro-Xaa carboxypeptidase | 5547 | ENSG00000137509 | PRCP |
| 5 | 3.4.11.9 | 0.00000 | Xaa-Pro aminopeptidase | 63929;7511;7512 | ENSG00000196236;ENSG00000108039;ENSG00000122121 | XPNPEP3;XPNPEP1;XPNPEP2 |
| 6 | 3.4.14.5 | 0.00000 | dipeptidyl-peptidase IV | 1803;54878;91039 | ENSG00000197635;ENSG00000074603;ENSG00000142002 | DPP4;DPP8;DPP9 |
| 7 | 3.4.23.1 | 0.00000 | pepsin A | 5222;643834;643847 | ENSG00000256713;ENSG00000229859;ENSG00000229183 | PGA5;PGA3;PGA4 |
| 8 | 3.4.24.18 | 0.00000 | meprin A | 4224 | ENSG00000112818 | MEP1A |
| 9 | 3.4.24.63 | 0.00000 | meprin B | 4225 | ENSG00000141434 | MEP1B |
| 10 | 3.4.17.1 | 0.00000 | carboxypeptidase A | 1357;1359;57094;93979 | ENSG00000091704;ENSG00000163751;ENSG00000165078;ENSG00000158525 | CPA1;CPA3;CPA6;CPA5 |
| 11 | 3.4.17.15 | 0.00000 | carboxypeptidase A2 | 1358 | ENSG00000158516 | CPA2 |
| 12 | 3.4.17.2 | 0.00000 | carboxypeptidase B | 1360 | ENSG00000153002 | CPB1 |
| 13 | 3.4.21.1 | 0.00000 | chymotrypsin | 1504;440387 | ENSG00000168925/ENSG00000285346;ENSG00000168928/ENSG00000284810 | CTRB1;CTRB2 |
| 14 | 3.4.21.70 | 0.00000 | pancreatic endopeptidase E | 10136;23436 | ENSG00000142789;ENSG00000219073 | CELA3A;CELA3B |
| 15 | 3.4.21.71 | 0.00000 | pancreatic elastase II | 51032;63036 | ENSG00000215704;ENSG00000142615 | CELA2B;CELA2A |
| 16 | 3.4.24.11 | 0.00000 | neprilysin | 4311;79258 | ENSG00000196549;ENSG00000142606/ENSG00000277131 | MME;MMEL1 |
| 17 | 3.5.1.1 | 0.00000 | asparaginase | 374569;80150 | ENSG00000166183;ENSG00000162174 | ASPG;ASRGL1 |
| 18 | 2.6.1.42 | 0.00000 | branched-chain-amino-acid transaminase | 586;587 | ENSG00000060982;ENSG00000105552 | BCAT1;BCAT2 |
| 19 | 4.1.1.17 | 0.00000 | ornithine decarboxylase | 4953 | ENSG00000115758 | ODC1 |
| 20 | 2.3.3.8 | 0.00000 | ATP citrate synthase | 47 | ENSG00000131473 | ACLY |
| 21 | 3.4.11.3 | 0.00000 | cystinyl aminopeptidase | 4012 | ENSG00000113441 | LNPEP |
| 22 | 3.4.11.7 | 0.00000 | glutamyl aminopeptidase | 2028 | ENSG00000138792 | ENPEP |
| 23 | 3.4.21.26 | 0.00000 | prolyl oligopeptidase | 5550 | ENSG00000085377 | PREP |
| 24 | 3.4.21.39 | 0.00000 | chymase | 1215 | ENSG00000092009 | CMA1 |
| 25 | 3.4.24.16 | 0.00000 | neurolysin | 57486 | ENSG00000123213 | NLN |
| 26 | 3.5.3.1 | 0.00000 | arginase | 383;384 | ENSG00000118520;ENSG00000081181 | ARG1;ARG2 |
| 27 | 3.4.17.20 | 0.00000 | carboxypeptidase U | 1361 | ENSG00000080618 | CPB2 |
| 28 | 2.6.1.19 | 0.00000 | 4-aminobutyrate---2-oxoglutarate transaminase | 18 | ENSG00000183044 | ABAT |
| 29 | 2.6.1.2 | 0.00000 | alanine transaminase | 2875;84706 | ENSG00000167701;ENSG00000166123 | GPT;GPT2 |
| 30 | 3.4.21.4 | 0.00000 | trypsin | 5644;5645;5646 | ENSG00000204983/ENSG00000274247;ENSG00000275896/ENSG00000282049;ENSG00000010438 | PRSS1;PRSS2;PRSS3 |
| 31 | 4.1.1.11 | 0.00000 | aspartate 1-decarboxylase | 339896 | ENSG00000144644 | GADL1 |
| 32 | 2.1.3.3 | 0.00000 | ornithine carbamoyltransferase | 5009 | ENSG00000036473 | OTC |
| 33 | 2.6.1.44 | 0.00000 | alanine---glyoxylate transaminase | 189;64902 | ENSG00000172482;ENSG00000113492 | AGXT;AGX2 |
| 34 | 4.3.1.19 | 0.00000 | threonine ammonia-lyase | 10993;113675 | ENSG00000135094;ENSG00000139410 | SDS;SDSL |
| 35 | 1.16.3.2 | 0.00000 | bacterial non-heme ferritin | 2495 | ENSG00000167996 | FTH1 |
| 36 | 2.7.11.2 | 0.00000 | [pyruvate dehydrogenase (acetyl-transferring)... | 5163;5164;5165;5166 | ENSG00000152256;ENSG00000005882;ENSG00000067992;ENSG00000004799 | PDK1;PDK2;PDK3;PDK4 |
| 37 | 2.6.1.51 | 0.00000 | serine---pyruvate transaminase | 189 | ENSG00000172482 | AGXT |
| 38 | 1.14.16.1 | 0.00000 | phenylalanine 4-monooxygenase | 5053 | ENSG00000171759 | PAH |
| 39 | 2.3.3.1 | 0.00000 | citrate (Si)-synthase | 1431 | ENSG00000062485 | CS |
| 40 | 2.6.1.64 | 0.00000 | glutamine---phenylpyruvate transaminase | 56267;883 | ENSG00000137944;ENSG00000171097 | KYAT3;KYAT1 |
| 41 | 7.2.2.13 | 0.00000 | Na+/K+-exchanging ATPase | 476;477;478;480 | ENSG00000163399;ENSG00000018625;ENSG00000105409;ENSG00000132681 | ATP1A1;ATP1A2;ATP1A3;ATP1A4 |
| 42 | 6.3.1.2 | 0.00000 | glutamine synthetase | 2752 | ENSG00000135821 | GLUL |
| 43 | 1.4.3.2 | 0.00000 | L-amino-acid oxidase | 259307 | ENSG00000104951 | IL4I1 |
| 44 | 6.3.4.2 | 0.00000 | CTP synthase (glutamine hydrolysing) | 1503;56474 | ENSG00000171793;ENSG00000047230 | CTPS1;CTPS2 |
| 45 | 2.1.4.1 | 0.00000 | glycine amidinotransferase | 2628 | ENSG00000171766 | GATM |
| 46 | 4.3.1.17 | 0.00001 | L-serine ammonia-lyase | 10993;113675 | ENSG00000135094;ENSG00000139410 | SDS;SDSL |
| 47 | 4.2.1.3 | 0.00001 | aconitate hydratase | 48;50 | ENSG00000122729;ENSG00000100412 | ACO1;ACO2 |
| 48 | 3.4.21.35 | 0.00002 | tissue kallikrein | 3816;3817 | ENSG00000167748;ENSG00000167751 | KLK1;KLK2 |
| 49 | 3.1.1.56 | 0.00003 | methylumbelliferyl-acetate deacetylase | 8824 | ENSG00000172831 | CES2 |
| 50 | 6.3.2.11 | 0.00003 | carnosine synthase | 57571 | ENSG00000172508 | CARNS1 |
| 51 | 7.2.2.10 | 0.00004 | P-type Ca2+ transporter | 27032;487;488;489;490;491;492;493;9914 | ENSG00000017260;ENSG00000196296;ENSG00000174437;ENSG00000074370;ENSG00000070961;ENSG00000157087;ENSG00000067842;ENSG00000058668;ENSG00000064270 | ATP2C1;ATP2A1;ATP2A2;ATP2A3;ATP2B1;ATP2B2;ATP2B3;ATP2B4;ATP2C2 |
| 52 | 2.6.1.13 | 0.00004 | ornithine aminotransferase | 4942 | ENSG00000065154 | OAT |
| 53 | 2.6.1.1 | 0.00008 | aspartate transaminase | 2805;2806 | ENSG00000120053;ENSG00000125166 | GOT1;GOT2 |
| 54 | 3.4.23.15 | 0.00013 | renin | 5972 | ENSG00000143839 | REN |
| 55 | 6.1.1.22 | 0.00013 | asparagine---tRNA ligase | 4677;79731 | ENSG00000134440;ENSG00000137513 | NARS;NARS2 |
| 56 | 3.5.1.89 | 0.00015 | N-acetylglucosaminylphosphatidylinositol deac... | 9487 | ENSG00000108474 | PIGL |
| 57 | 2.6.1.45 | 0.00015 | serine---glyoxylate transaminase | 189 | ENSG00000172482 | AGXT |
| 58 | 2.6.1.5 | 0.00027 | tyrosine transaminase | 6898 | ENSG00000198650 | TAT |
| 59 | 3.4.15.1 | 0.00047 | peptidyl-dipeptidase A | 1636 | ENSG00000159640 | ACE |
| 60 | 3.4.19.5 | 0.00069 | beta-aspartyl-peptidase | 80150 | ENSG00000162174 | ASRGL1 |
| 61 | 3.4.16.5 | 0.00072 | carboxypeptidase C | 5476 | ENSG00000064601 | CTSA |
| 62 | 2.6.1.16 | 0.00084 | glutamine---fructose-6-phosphate transaminase... | 2673;9945 | ENSG00000198380;ENSG00000131459 | GFPT1/GFPT2 |
| 63 | 6.3.4.16 | 0.00095 | carbamoyl-phosphate synthase (ammonia) | 1373 | ENSG00000021826 | CPS1 |
| 64 | 4.4.1.13 | 0.00098 | cysteine-S-conjugate beta-lyase | 56267;883 | ENSG00000137944;ENSG00000171097 | KYAT3;KYAT1 |

**MATERIAL AND METHODS**

**Antibodies and reagents**. The primary antibodies used were: a) rabbit polyclonal – giantin (Novus Biologicals, NBP2-22321 and Abcam: ab24586 and ab93281), S1P (ab224618 and ab59870), S2P (ab244279 and ab196797), ATF6 (ab203119), GRP78 (ab21685), Lamin B1 (ab16048), GCC185 (ab128173), GCC185 (Thermo Fisher Scientific, A303-569A-T), GCC185 (Novus Biologicals, NBP2-04024), GCC88 (LifeSpan BioSciences, LS‑C678055), Golgin-245 (ab122250) ; b) rabbit monoclonal – TMF (ab151702), GM130 (ab52649); c) mouse monoclonal – S1P (LifeSpan BioSciences, LS-C308907 and Abnova, H00008720-M07), S2P (Santa Cruz Biotechnology, sc-293341), HSP70 (ab2787), ATF6 (ab122897), β-actin (Sigma, A2228), giantin (Abcam, ab37266); d) mouse polyclonal – GM130 (Abcam, ab169276). The secondary antibodies (Jackson ImmunoResearch) were: a) HRP-conjugated donkey anti-rabbit (711-035-152) and donkey anti-mouse (715-035-151) for W-B; b) donkey anti-mouse Alexa Fluor 488 (715-545-150) and 595 (715-585-150), anti-rabbit Alexa Fluor 488 (711-545-152) and 594 (711-585-152) for immunofluorescence. Thapsigargin (Calbiochem) was dissolved in DMSO immediately before use. Thapsigargin was added to the cultured cells at a final concentration of 1 μM, which was followed by incubation at 37°C for 4 h. NEM (N-ethylmaleimide), obtained from Thermo Fisher Scientific, was dissolved in water, resulting in a 2 mM concentration, then applied to the cell pellet prior to the cell lysis buffer. The remaining chemicals and reagents, including methanol and DMSO, were of MS-grade/analytical grade and purchased from Sigma.

**Cell culture and EtOH treatment**. LNCaP, 22Rv1, and PC-3 cells were purchased from ATCC. Cells were grown in phenol red-free RPMI medium with 11 mM glucose, 10% FBS, 2mM glutamine, non-essential amino acids, and 100U/ml of Penicillin plus Streptomycin. Given their androgen responsiveness, cells were treated with 10 nM dihydrotestosterone (DHT). The immortalized human prostate cell line RWPE-1 was from ATCC, and cells were grown in Keratinocyte serum-free medium, which contains 50 µg/ml of BPE and 5 ng/ml EGF, plus an antibiotic/antimycotic mixture (Penicillin, 100 U/ml, Streptomycin 100 µg/ml and Fungizone, 25 µg/ml). Twenty-four hours after seeding cells (at ∼75% confluence), the culture media was replaced with one containing 50 mM EtOH and incubated for an additional 96 h. The medium was replaced every 48 h to maintain a constant EtOH concentration. Control cells were seeded simultaneously as treated cells and maintained in the same medium; EtOH was replaced by the appropriate volume of medium to maintain similar caloric content.

**Soft agar assay for colony formation**. For the anchorage-independent assay, LNCaP cells were seeded in 60 mm Petri dishes, and a base of 0.5% agar +1X RPMI + 10% FBS was prepared together with a top of 0.3% agar + 2X RPMI + 20% FBS. Cells were incubated at 37° C in a humidified incubator for 10 days and treated with or without EtOH. After 10 days, colonies were observed at EVOS AMF-4300 microscope (AMG) microscope. The representative phase-contrast digital images were captured with a 20x lens, and the number of colonies was quantified in 20 randomly selected areas. In order to count all the colonies seeded at the different levels of agar, the focal depth of the microscope was adjusted manually for each area.

**Cell migration assay**. For assay cell migration, 2×104 cells suspended in 200 µl RPMI-1640 medium without FBS were seeded onto the fibronectin-coated polycarbonate membrane of a Transwell® insert (Corning). A volume of 600 µl RPMI-1640 with 10% FBS was added as a chemoattractant in the lower chamber. Following incubation for 72 h at 37°C in a 5% CO2 atmosphere, the Transwell insert was washed with PBS, and the cells on the top surface of the insert were removed with a cotton swab. Cells adhering to the lower surface were fixed with ice-cold 100% methanol for 10 min, washed with PBS three times, stained with DAPI, and finally air-dried. The migrated cells were counted using the EVOS AMF-4300 microscope (AMG) microscope, and the representative phase-contrast digital images captured with a 20x lens.

**Immunohistochemistry**. The 4 µm paraffin sections were processed using BOND-MAX Fully automated IHC instrument. Samples were deparaffinized, rehydrated in decreasing concentrations of ethanol, followed by heat (95ºC) induced epitope retrieval in 10 mM sodium citrate buffer, pH 6, and staining with appropriate primary Abs. Next, samples were incubated with BOND Polymer Refine Detection system. The control samples were treated analogously except incubation with primary antibodies. Images were analyzed using the Aperio ImageScope (Leica biosystems). The intensity of signals were counted as a sum of the total intensity of weak positive, positive, and strong positive. The ratio total intensity/area (mm2) was compared between each group of samples.

**Confocal immunofluorescence microscopy**. The immunostaining of cells was performed by the methods described previously 47. Slides were examined under a Zeiss LSM 800 Zeiss Airyscan Microscope performed at the Advanced Microscopy Core Facility of the University of Nebraska Medical Center. Fluorescence was detected with a fixed exposure time, using an emission filter of a 505 to 550 nm bandpass for green and a 575 to 615 nm bandpass for red. Images were analyzed using ZEN 2.3 SP1 software and IMARIS versions 7.2.2–7.6.0 (Bitplane Scientific). Confocal images from the same series of experiments were acquired with identical imaging parameters. For some figures, image analysis was performed using Adobe Photoshop and Fiji. Statistical analysis of colocalization was performed by Fiji, calculating the Pearson correlation coefficient 48.

**Three-dimensional structured illumination (3D-SIM) microscopy and image analysis**. SIM imaging of intracellular organelles was performed at the Advanced Microscopy Core Facility of the University of Nebraska Medical Center using a Zeiss ELYRA PS.1 super-resolution scope (Carl Zeiss Microscopy, Germany), which is equipped with a PCO.Edge 5.5 camera and a Plan-Apochromat 63×1.4 oil objective. Optimal grid sizes for each wavelength were chosen according to Zeiss’s recommendations. For 3D-SIM, stacks with a step size of 110 nm were acquired sequentially for each fluorophore, and each fluorescent channel was imaged with three pattern rotations with three translational shifts. The final SIM image was created using modules built into the Zen Black software suite accompanying the imaging setup. Analyses were undertaken on 3D-SIM data sets in 3D using IMARIS versions 7.2.2–7.6.0 (Bitplane Scientific). The 3D mask was obtained by applying a Gaussian filter to merged channels, thresholding to remove low-intensity signals, and converting the obtained stack into a binary file that mapped all voxels of interest for coefficient calculation. For colocalization studies, the IMARIS “Colocalization Module” was used based on the nearest neighbor distances to consider the Nyquist limited resolution, which in our case was around ~94 nm 49. To avoid subjectivity, all thresholds were automatically determined using algorithms by Costes et al. 50 and based on the exclusion of intensity pairs that exhibit no correlation. Colocalization was determined by Pearson coefficient, which represents a correlation of channels inside colocalized regions.

**AFM imaging and image analysis**. GCC185 was isolated from LNCaP and PC-3 cells using anti-GCC18 Ab coupled Epoxy beads (Dynabeads M-450) (Thermo Fisher Scientific) according to the manufacturer's recommendations. Eluted IP samples were isolated using Millipore UFC500324 Amicon Ultra Centrifugal Filters and then dissolved in PBS for pH neutralization and treated with 2% of β-mercaptoethanol. Freshly cleaved mica was modified with 1-(3-aminopropyl)-silatrane (APS) as previously described 51. 5-10 µL of the sample were deposited on the piece of APS mica and after 2 min incubation, samples were rinsed briefly with several drops of deionized water and dried with a gentle flow of argon. Images were collected with the MultiMode Nanoscope IV system (Bruker Instruments, Santa Barbara, CA) in Tapping Mode at ambient conditions. Silicon probes RTESPA-300 (Bruker Nano Inc., CA, USA) with a resonance frequency of ~300 kHz and a spring constant of ~40 N/m were used for imaging at a scanning rate of about 2.0 Hz. Images were processed using the FemtoScanOnline software package (Advanced Technologies Center, Moscow, Russia). Height and diameter were obtained with Enum Features semi-automatic image analysis. The volume of protein was approximated as hemisphere and calculated as V= *H/6 (3/4D2 + H2), where H is the height and D is diameter as previously described 52.

**In situ Proximity Ligation Assay (PLA)**. The assay was performed using the Duolink kit (Sigma) according to the manufacturer’s protocol. The mouse monoclonal – S1P (LifeSpan BioSciences, LS-C308907) and S2P (Santa Cruz Biotechnology, sc-293341) Abs were used in combination with rabbit polyclonal GCC185 Ab (Abcam, ab128173). After incubation with primary Abs, the tissue section slides were exposed to oligonucleotide-conjugated anti-mouse minus and anti-rabbit plus proximity ligation assay secondary probes followed by ligation and amplification of their oligonucleotides. Subsequent PLA signals (red fluorescent spots) were captured using the EVOS M5000 Imaging System (Thermo Fisher Scientific, USA) and processed by Image J/Fiji. First, we selected the sample with the lowest PLA signal (Fig. S2, low panel). Second, the areas of nonspecific signal (Fig. S2, white arrowheads, step #1) were dissected (Fig. S2, step #2), then the cut image was converted to an 8-bit image (Fig. S2, step #3). Finally, using the “Threshold” module of Fiji, the threshold was standardized to delineate the labeled structures (Fig. S2, step #4) and then applied to all images from individual tissue sections, which were processed the same way as described (Fig. S2, top panel).

**NMR data collection and analysis**. For cell media samples, a total of 1 mL of media was collected, immediately frozen with liquid nitrogen, and stored at -80 °C until NMR sample preparation. For cell extract samples, attached cells (to the plates) were washed twice with 5 mL of PBS to remove debris, then lysed and quenched with 1 mL of pre-chilled methanol at −20 °C. To detach (from the plate), cells were incubated at -80 °C for 15 min with detachment confirmed using microscopy. The cell suspension was then detached and transferred in methanol to a 2 mL microcentrifuge tube and centrifuged for 5 min at 15,000 × g at 4 °C to remove pellet debris. The resulting supernatant was transferred to a new 2 mL microcentrifuge tube, and the extraction was repeated twice more to improve overall efficiency – first with 0.5 mL of 80%/20% methanol/water pre-chilled at −20 °C and second with 0.5 mL of ice-cold water – collecting the supernatant after each round (combining all extractions). Finally, methanol solvent was removed/evaporated using a SpeedVac / rotary evaporator, flash-frozen in liquid nitrogen, freeze-dried using a lyophilizer, and stored at -80 °C until NMR sample preparation. For 2D NMR preparation, lyophilized cell-free lysates were suspended in 550 μL of 100% 50 mM D2O phosphate buffer at pH 7.2 with 500 μM TMSP-d4 used as a chemical shift and concentration reference. Samples were then centrifuged at 14,000 × g for 10 min to remove any particulates and transferred to a 4″ 5 mM SampleJet NMR tube with a RAININ XLS pipette. Finally, each sample was added to a 96-well plate; the SampleJet configuration was equilibrated to 4 °C to prevent metabolite degradation. All NMR spectra were collected at 298K using a Bruker AVANCE III-HD 700 MHz spectrometer equipped with a 5 mm quadruple resonance QCI-P cryoprobe with z-axis gradients. Automated NMR data collection was facilitated using a SampleJet sample changer, automatic tuning and matching accessory, and ICON-NMR software. The 2D 1H- 13C HSQC spectra were acquired in the DQD acquisition mode with 8 scans, 16 dummy scans, and a 1.5 s relaxation delay. The spectra were collected with 2048 data points with a spectral width of 11,160 Hz in the direct dimension and 128 data points with a spectral width of 29,059 Hz in the indirect dimension. 2D NMR data processing and analysis were carried out using NMRpipe 53 and NMRViewJ 54. A data matrix consisting of relative metabolite peak intensities (rows) and biological replicates (columns) was produced from the 2D 1H-13C HSQC data set. Metabolites were assigned using PRIMe SpinAssign (http://dmar.riken.jp/spinassign) 55,56, the Human Metabolome Database (HMDB) (http://www.hmdb.ca) 57 and the BioMagResBank (BMRB) (www.bmrb.wisc.edu) 58 by matching reference chemical shifts from the database to the experimental spectra using a peak-error tolerance of 0.08 ppm and 0.25 ppm for 1H and 13C chemical shifts, respectively 57. The data matrix generated was used for statistical and pathway analysis.

**Isolation of Golgi membrane fractions by sucrose gradient centrifugation**. Golgi membrane fractions were isolated using methods described previously 47. Cells from ten-to-twelve 75 cm² cell culture flasks were harvested with PBS containing 0.5x protease and phosphatase inhibitors (1.2 ml per flask). After centrifugation for 5 min at 1000 rpm at 4°C, the pellet was resuspended in 3 ml of homogenization buffer (0.25 M sucrose, 3 mM imidazole, 1 mM Tris-HCl; pH 7.4, 1 mM EDTA). Cells were homogenized by drawing ~ 30 times through a 25-gauge needle until the ratio between unbroken cells and free nuclei became 20%:80%. The postnuclear supernatant was obtained by centrifugation at 2,500 rpm at 4°C for 3 min, and then the supernatant was adjusted to 1.4 M sucrose by the addition of ice-cold 2.3 M sucrose in 10 mM Tris-HCl (pH 7.4). Next, 1.2 ml of 2.3 M sucrose at the bottom of the tube was overlaid with 1.2 ml of the supernatant adjusted to 1.4 M sucrose followed by sequential overlay with 1.2 ml of 1.2 M and 0.5 ml of 0.8 M sucrose (10 mM Tris-HCl, pH 7.4). Gradients were centrifuged for 3 h at 38,000 rpm (4°C) in an SW40 rotor (Beckman Coulter). The turbid band, at the 0.8 M/1.2 M sucrose interface containing the Golgi membranes, was harvested in ~500 µl aliquot by syringe puncture. The fraction at a concentration of ~1.0-1.4 mg protein/ml was used for the experiments mentioned in the Results section. To prepare the Golgi heavy and light fractions, the isolated Golgi membranes were subjected to centrifugation on 0.25/0.6/0.8 M Tris-HCl (pH 7.4) sucrose gradients51. The Golgi membranes were adjusted to 2.2 ml of 0.8 M sucrose and loaded at the bottom of the tube, followed by 1.1 ml of 0.6 M sucrose and then 550 µl of 0.25 M sucrose. The gradients were centrifuged for 4 h at 42,000 rpm (4°C) in a SW60 rotor (Beckman Coulter). The turbid band between 0.6 M and 0.8 M sucrose, which contains the Golgi heavy fraction corresponding to cis-medial-Golgi, and the 0.25 M/0.6 M sucrose interface, which contains Golgi light fraction corresponding to the trans-Golgi, were collected. Giantin and TGN46 served as markers for cis-medial-59 and trans-Golgi 60, respectively.

**Isolation of microsomal fraction**. Isolation of microsomes was performed using the Endoplasmic Reticulum Isolation Kit (Sigma) according to the manufacture’s protocol. Briefly, cells were suspended in Hypotonic Extraction Buffer and incubated for 20 minutes at 4°C to allow the cells to swell. After the centrifugation at 6,000 × g for 5 minutes and homogenization, the homogenate was centrifuged at 1,000 × g for 10 minutes (4ºC). The obtained postnuclear supernatant was centrifuged at 12,000 × g for 15 minutes (4ºC), and supernatant (post mitochondrial fraction) was further subjected to isolation of the rough endoplasmic reticulum (RER) using precipitation by 8 mM CaCl2.

**Immunoprecipitation (IP), plasmid constructions, and transfection**. To identify the proteins within the complexes pulled down by IP, confluent cells grown in a T75 flask were washed three times with 6 ml of PBS, harvested by trypsinization, and neutralized using the soybean trypsin inhibitor containing a 2x weight of trypsin. IP steps were performed using Dynabeads M-270 Epoxy beads. Following the antibody's immobilization, the coupled beads were incubated with cell lysate overnight (4ºC), and the IP protein complexes were eluted from the beads. All cell lysate samples for IP experiments were normalized by appropriate proteins. To determine whether the target protein was loaded evenly, input samples were preliminarily run on a separate gel with different dilutions of control vs. treated samples, then probed with anti-target protein Abs. The intensity of the obtained bands was analyzed using Fiji software, samples with an identical intensity were subjected to IP. GCC2 (GCC185), ATF6, and scrambled on-targetplus smartpool siRNAs were purchased from Santa Cruz Biotechnology. All products consisted of pools of three target-specific siRNAs. Cells were transfected with 100 nM siRNAs using Lipofectamine RNAi MAX reagent (Life science technologies). Transfection of ATF6 shRNA (Santa Cruz Biotechnology) was performed according to the manufacturer’s recommendations; 48 hours post-transfection, the medium was replaced with fresh medium containing puromycin at a 1 µg/ml concentration. pEGFP-ATF6 was a gift from Ron Prywes (Addgene plasmid # 32955 ; http://n2t.net/addgene:32955 ; RRID:Addgene_32955); hGCC185 (Myc-DDK-tagged) plasmid was obtained from Origene (cat. # RC219795).

**Quantitative gene expression analysis by qRT-PCR**. RNA from cultured prostate cells were isolated by TRI-REAGENT (MRC Inc.) according to the manufacturer’s instruction. To prepare cDNA, 2 μg RNA was used in a 20 μl reaction mixture using a Verso Reverse Transcriptase Kit (Thermo Scientific) as follows: 5 min at RT, 60 min at 42°C, and 2 min at 95°C. Quantitative real-time PCR of the mRNAs from the genes which encode GRP78, calreticulin, and GRP94 was performed in a 10 μl reaction volume in a 96-well plate using 2 μl of diluted (1:1) cDNA with SYBR Premix Ex Taq™ (Takar Bio Inc.) on a Mastercycler ep gradient realplex system (Eppendorf). The PCR conditions included 1 cycle at 95°C for 2 min followed by 45 cycles at 95°C for 15 s, 60°C for 15 s, and 72°C for 45 s. The data were analyzed using Eppendorf realplex software, version 1.5 (Eppendorf). Glyceraldehyde-3-phosphate dehydrogenase (GAPDH) was used as a control reaction for each sample. Relative fold differences in transcript expression were determined using the following comparative CT method: 2-[ΔCt (Target)]×100 where ΔCt=Ct(Target) – Ct (GAPDH) as described previously 19. The results were expressed as the fold relative to that (100 %, 1 fold) of GAPDH and plotted as mean expression ± SD.

**Quantification and statistical analysis**. Statistical analyses were performed using Microsoft Excel and GraphPad Prism 8.0 software (GraphPad). Statistical parameters are expressed as the mean ± SD or medians (min – max). Multiparametric analysis was done using the Wilcoxon rank sum test between all condition with Bonferonni-Hochberg multiple test correction. Mann-Whitney test or Student’s t test, unpaired, two-sided, two-tailed, one-way or two-way analysis of variance (ANOVA) were used to analyze data as indicated. The corresponding sample size and p-values are reported in the Figures and Figure legends.
